# From Single to Multi‐Material 3D Printing of Glass‐Ceramics for Micro‐Optics

**DOI:** 10.1002/smtd.202401809

**Published:** 2025-02-03

**Authors:** Joel Arriaga‐Dávila, Cristian Rosero‐Arias, Dirk Jonker, Margoth Córdova‐Castro, Josua Zscheile, Robert Kirchner, Alan Aguirre‐Soto, Robert Boyd, Israel De Leon, Han Gardeniers, Arturo Susarrey‐Arce

**Affiliations:** ^1^ Department of Chemical Engineering Mesoscale Chemical Systems MESA+ Institute University of Twente PO Box 217 Enschede 7500 AE The Netherlands; ^2^ School of Engineering and Sciences Tecnológico de Monterrey Eugenio Garza Sada 2501 Monterrey NL 64849 Mexico; ^3^ Department of Physics University of Ottawa Ottawa Ontario K1N 6N5 Canada; ^4^ HETEROMERGE GmbH Gostritzer Str. 61 01217 Dresden Germany; ^5^ Center for Advancing Electronics Dresden TU Dresden, Helmholtzstraße 18 01069 Dresden Germany; ^6^ Department of Physics University of Rochester Rochester NY 14627 USA; ^7^ School of Electrical Engineering and Computer Science University of Ottawa Ottawa Ontario K1N6N5 Canada; ^8^ ASML Netherlands B.V. De Run 6501 DR Veldhoven 5504 The Netherlands

**Keywords:** 3D printing, additive manufacturing, glass‐ceramics, micro‐optics, multi‐material

## Abstract

Feynman's statement, “There is plenty of room at the bottom”, underscores vast potential at the atomic scale, envisioning microscopic machines. Today, this vision extends into 3D space, where thousands of atoms and molecules are volumetrically patterned to create light‐driven technologies. To fully harness their potential, 3D designs must incorporate high‐refractive‐index elements with exceptional mechanical and chemical resilience. The frontier, however, lies in creating spatially patterned micro‐optical architectures in glass and ceramic materials of dissimilar compositions. This multi‐material capability enables novel ways of shaping light, leveraging the interaction between diverse interfaced chemical compositions to push optical boundaries. Specifically, it encompasses both multi‐material integration within the same architectures and the use of different materials for distinct architectural features in an optical system. Integrating fluid handling systems with two‐photon lithography (TPL) provides a promising approach for rapidly prototyping such complex components. This review examines single and multi‐material TPL processes, discussing photoresin customization, essential physico‐chemical conditions, and the need for cross‐scale characterization to assess optical quality. It reflects on challenges in characterizing multi‐scale architectures and outlines advancements in TPL for both single and spatially patterned multi‐material structures. The roadmap provides a bridge between research and industry, emphasizing collaboration and contributions to advancing micro‐optics.

## Graphical abstract

This article explores advancements in two‐photon lithography (TPL) for fabricating high‐refractive‐index micro‐optics. It examines single and multi‐material 3D architectures in glass and ceramics (GLACEs), highlighting photoresin customization, critical fabrication conditions, and cross‐scale structural and optical characterization. By bridging research and industry, it outlines opportunities for fully integrated micro‐optical components.

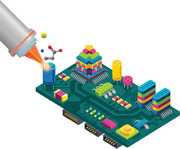

## 1 Introduction

Pursuing 3D architectures has stimulated the development of advanced techniques permitting the shaping of matter across length scales. On one side of the scale, you can find yourself walking over the 3D‐printed steel bridge in Amsterdam,^[^ ^1^ ^]^ while at the other end of the scale, the naked human eye cannot resolve the smallest printed micro‐optical architectures, which are smaller than human hair.^[^ ^2^, ^3^ ^]^ The miniaturization of such microarchitectures with nanometric feature sizes using additive manufacturing (AM), also known as 3D printing, has acquired enormous relevance in optics. AM is expected to transform the micro‐optical industry in response to the growing demand for miniaturized devices,^[^ ^4^, ^5^ ^]^ unlike current subtractive manufacturing (SM),^[^ ^6^ ^]^ which slices objects from a solid block, limiting shape and geometry diversity.

AM builds objects layer by layer from scratch, offering 3D free‐form potential. The AM approach enables limitless geometries tailored for specific optical functions, such as spatially distributed information for data storage.^[^ ^7^, ^8^ ^]^ However, to manufacture emerging micro‐optical devices, precise printing control in the micrometer range is necessary.^[^ ^9^, ^10^, ^11^ ^]^ Modern AM offers various 3D printing technologies with distinct working principles categorized into seven groups^[^ ^12^ ^]^: Binder jetting (BJT),^[^ ^13^ ^]^ Powder bed fusion (PBF),^[^ ^14^ ^]^ Directed energy deposition (DED),^[^ ^15^ ^]^ Material jetting (MJT),^[^ ^16^ ^]^ Material extrusion (MEX),^[^ ^17^ ^]^ Sheet lamination (SHL),^[^ ^18^ ^]^ and Vat photopolymerization (VPP),^[^ ^19^ ^]^ highlighting AM's diversity. Nevertheless, due to their high‐resolution capabilities, micro‐optics research often aligns with PBF, MJT, or VPP. More specifically, VPP‐based methods like projection micro‐stereolithography (PµSL) and TPL are preferred. PµSL is a combined technology based on stereolithography (SL) and digital light processing (DLP). SL involves light irradiation on a photosensitive resin (photoresin) contained in a vat. The laser reproduces the aimed geometry and features by scanning layer by layer. As for DLP, the approach also cross‐links photocurable material. However, the method integrates a digital micromirror device (DMD) that projects light as a mask, translating into patterning a total layer without scanning the x and y‐axis.

Compared to PµSL, the uniqueness of TPL is manifested by its truly sub‐diffraction‐limit printing resolution.^[^ ^3^, ^20^ ^]^ TPL relies on the local photopolymerization of photoresins upon a two‐photon absorption (TPA) event stimulated by focused and pulsed femtosecond laser radiation. The position of the focal point can be moved through the photoresin to write the desired 3D photopolymer architectures directly. Upon exposure, the non‐polymerized liquid photoresin is washed away in the development step, and the printed architecture with ultra‐high resolution is achieved.^[^ ^20^ ^]^

TPL offers versatility across various target materials. Cutting‐edge printable materials accessible through photocurable resin formulations have been tailored for polymers, composites, glasses, and ceramics.^[^ ^21^, ^22^, ^23^ ^]^ Among them, high‐refractive‐index materials, like glass and ceramics, can overcome current polymer‐based TPL‐printed optical elements' drawbacks, e.g., chemical, mechanical, and long‐term stability under harsh environments.^[^ ^24^ ^]^ Although glass and ceramic (GLACE) photoresins are at the early stage, the photoresin can be carefully tuned to maximize inorganic loading or loadings. Maximizing inorganic payloads in an organic matrix imposes miscibility issues, compromising photoresin's desired characteristics like stability. Hence, inorganic and organic components should blend perfectly and remain stable over time, preferably with a polar environment, to enhance compatibility with often required inorganic salts.^[^ ^25^ ^]^

Under the most favorable circumstances, with photoresin characteristics meticulously tailored for GLACE production, the next step is printing to produce a pre‐GLACE replica, which is thermally treated in a subsequent step.^[^ ^24^ ^]^ The thermal step imposes other challenges, particularly to photoresins with poor inorganic payloads, which can compromise the microarchitecture's mechanical stability upon annealing. Defects can also accompany pre‐GLACE thermal transformation. Defects typically overlooked within the GLACE community can be encountered over multiple mesoscopic levels. For example, microscopic structural defect‐like surface roughness, internal void formation within the GLACE body, or defects encountered at the atomic level, such as vacancies, which can contribute to unwelcome emissive fluorescent signals compromising the quality of the optical element.^[^ ^24^ ^]^

Mitigation strategies to control defect levels within printed GLACEs should account for comprehensive studies on the thermodynamic equilibrium of the target crystal phase. An example of defect control in a material is silicon,^[^ ^26^ ^]^ in which defect and doping levels can be modulated. However, GLACE defects might be more challenging to control. Although unwelcome imperfections are prevalent, a way forward is taming defects by using defect engineering aspects to unlock defect manipulation, which can produce semiconductor devices with electrical and optical functionalities. Furthermore, a relatively unexplored terrain, like multi‐material spatial patterning, is discussed. We review such approaches, particularly emphasizing spatial multi‐material patterning.^[^ ^24^, ^27^ ^]^ Specifically, this includes both multi‐material integration within the same architectures and the use of different materials for distinct architectural features within a system. Such level advancements can close the loop to achieve spatially dissimilar GLACE compositions embedded within architectures applied in micro‐optics and other industries like semiconductors.^[^ ^24^ ^]^

Furthermore, we reflect upon analytical technologies, since GLACE chemical properties can be challenging to understand to the finest detail due to the limited analytical technology capable of thoroughly interrogating microscopic structures. Hence, analytical technology (in other words, metrology) should sufficiently advance to understand GLACE properties. Recurring challenges within metrology, e.g., bridging the gap between length scales, remain. Under the most favorable circumstances, GLACE examination should collect optical, chemical, and structural information within a few hundred microns and go down to atomic levels. An assessment of analytical methods that could enable chemical and structural interrogation is provided, and areas of opportunity are proposed.

Having laid the groundwork for the rational design of photoresins, GLACE production, and the requirements to understand GLACE micro‐optical components' chemical and structural information, GLACE integration within an optical system is a missing link. Hence, exploring ways of integrating optical grade GLACEs into optical systems is crucial to benchmark their applicability. Particular attention is given to pathways for integrating GLACE components with low‐temperature processes relevant in transitioning to the semiconductor industry.^[^ ^22^ ^]^ GLACEs integration might include reconfigurable photonic integrated chips (R‐PICs), fiber optic communication systems, quantum optics, and micro‐opto‐electro‐mechanical systems (MOEMS).^[^ ^28^ ^]^ Among the previously mentioned applications, a noteworthy example is the critical significance of TPL in fabricating micro‐optical elements directly onto active devices, such as R‐PIC.^[^ ^29^ ^]^ This capability could enable automated and efficient coupling of multi‐GLACE elements, offering flexibility and performance in micro‐optical systems with spatially patterned compositions. By thoughtfully considering the above optical technologies, we expect GLACE elements to advance next‐generation micro‐optical systems.

In short, this review encompasses diverse micro‐optical devices, providing insights into the past, present, and future TPL advancements for single and multi‐material patterning and highlighting the evolving landscape of micro‐optics manufacturing. For such endeavors, the review delves into advancements enabling (multi‐material) printing in micro‐optics manufacturing. It highlights the role of tailored TPL material formulations that facilitate defect‐free optical‐grade glass‐ceramic components. Characterization methods bridging micrometer to nanometer scales are assessed to understand structural, chemical, and optical properties within GLACEs. Prospect applications for optical components, including their integration into micro‐optical active systems, are explored. A road map of the steps required to bridge academia and the transitioning industry is provided.

## 2 Additive Manufacturing Meets the Optical Length Scale

Over the past two decades, AM, also known as 3D printing,^[^ ^4^, ^30^ ^]^ has revolutionized the micro‐optical industry due to the high‐priority demand for miniaturized devices, where their high‐precision 3D fabrication capabilities stand out over SM alternatives. SM relies on machining (e.g., cutting, milling, and drilling) a starting block to shape it to the desired geometry,^[^ ^6^ ^]^ whereas AM builds a 3D object, typically layer by layer, from scratch. Besides its 3D freeform shaping potential, AM offers concrete advantages,^[^ ^7^, ^8^ ^]^ such as structural design freedom leading to limitless arbitrary geometries shaped to fulfill optical functionality, which can motivate spatial information storage. The latest assets are of utmost importance as 3D multi‐scale fabrication approaches can circumvent the need for multi‐processing, alleviating the path for mask‐free technologies typically encountered in the semiconductor industry, which can open the path to fast iteration and prototyping. These benefits allow AM to be established as a leading technology due to the potential for enriching the fabrication of optical devices with definite precision at the microscale with computer‐aided design (CAD) control. AM‐CAD can unlock new possibilities to integrate tailor‐made complex optical elements within microscale devices such as MOEMS, optofluidic microdevice systems, optical sensors, or emerging quantum technologies.^[^ ^28^ ^]^

Modern AM methods and techniques comprise a variety of 3D printing technologies with contrasting working principles, which limits their strict comparability. Standard classifications help delineate the landscape of cutting‐edge 3D printing technologies. According to the ISO/ASTM 52900:2021 standard,^[^ ^12^ ^]^ AM processes can be categorized into seven distinct groups. First, BJT relies on selectively depositing a liquid bonding agent in a powder bed,^[^ ^13^ ^]^ which results in bonded powder particles creating the final architecture. Second, PBF utilizes energy‐sintering sources (i.e., laser sintering and electron beam melting) to fuse powder locally within a bed layer,^[^ ^14^ ^]^ leading to the 3D object. Third, DED depends on focused thermal energy (e.g., laser, electron beam, plasma arc),^[^ ^15^ ^]^ but solid materials are dispensed selectively for melting. Fourth, in MJT, feedstock droplets of photoresin and wax are jetted and solidified when exposed to ultraviolet (UV) light or other curing methods.^[^ ^16^ ^]^ Fifth, MEX utilizes a nozzle or extruder to dispense fused materials onto a build platform,^[^ ^17^ ^]^ where layers are soldered together when cooling down or solidifying. Sixth, SHL employs thin material sheets coated with thermally activated glue,^[^ ^18^ ^]^ which incorporates a laser beam to cut the contour of the 3D shape when bonding/gluing layers. Seventh, VPP implements a liquid photocurable resin contained in a vat,^[^ ^19^ ^]^ where the architecture is selectively cured or solidified layer by layer using UV light or laser beams.

These categories and their specific derivative 3D printing technologies can be tailored for the comprehensive fabrication of optical devices. However, to fulfill the manufacture of emerging micro‐optical devices, precise printing control in the micrometer range is essential. In this direction, micro‐optics research often aligns with PBF, MJT, or VPP processes due to their advantageous high‐resolution capabilities.^[^ ^31^ ^]^ More specifically, VPP‐based methods like PµSL and TPL are preferred. PµSL^[^ ^32^ ^]^ is a combined technology based on SL and DLP. SL involves light irradiation on a photosensitive resin (photoresin) contained in a vat to scan features layer by layer, and DLP cross‐links photocurable materials while integrating a DMD that projects light as a mask, translating into patterning a total layer without scanning in the x and y‐axis. Thus, PµSL exploits the usage of DMD to mask microscale features while exposing them to light.

TPL, a direct laser writing‐based technology, uses a focused femtosecond laser beam to induce cross‐linking of the photoresin.^[^ ^3^ ^]^ Unlike most VPP techniques that use single‐photon absorption (SPA), TPL requires simultaneous TPA to solidify the material, enabling precise details and complex 3D architectures. More specifically, TPA enables a voxel‐by‐voxel approach instead of a layer‐by‐layer polymerization inherent to SPA, where the voxel, namely a 3D representation of 2D pixels, enables resolution beyond the diffraction limit.^[^ ^33^ ^]^ In addition, micro‐selective laser sintering (µ‐SLS, PBF‐based method) and direct inkjet writing/jetting (DIW or 3D inkjet printing, MJT‐based method) are also feasible options. µ‐SLS, just like standard selective laser sintering (SLS), utilizes a highly energetic laser, which locally heats and sinters the powder material.^[^ ^34^, ^35^ ^]^ Nevertheless, the previously mentioned method implements a particle bed formation/spreader setup to create submicron powder particles and an optical system to ensure the expected sintering micron resolution. DIW uses an inkjet printhead or nozzles to deposit material droplets or continuous‐flow liquid filaments to pattern an object.^[^ ^36^ ^]^ Then, the ink undergoes solidification with alternative processes like solvent evaporation, gelation, UV‐curing, or energy‐induced phase change. In literature, we can find diverse reports of micro‐optical elements fabricated with these methods, such as microlenses,^[^ ^28^, ^37^, ^38^ ^]^ diffractive optical elements (DOEs),^[^ ^39^, ^40^ ^]^ micro‐gratings,^[^ ^41^, ^42^ ^]^ optical fibers,^[^ ^43^, ^44^ ^]^ and waveguides.^[^ ^45^, ^46^, ^47^, ^48^ ^]^

**Figure 1:**
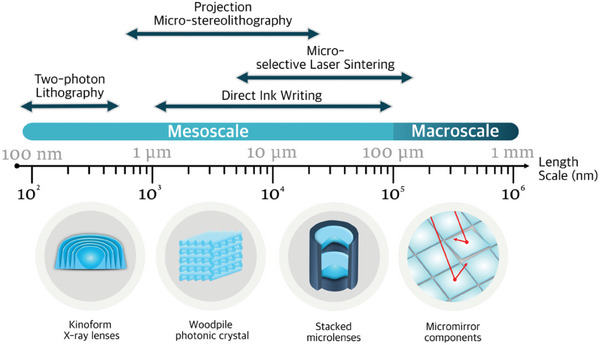
Schematic depiction of the feature size resolution range of TPL, PµSL, µ‐SLS, and DIW, along with illustration devices like Kinoform X‐ray lenses,^[^ ^63^ ^]^ woodpile photonic crystals,^[^ ^67^, ^68^ ^]^ stacked microlenses,^[^ ^28^, ^69^ ^]^ and micromirror components^[^ ^70^, ^71^ ^]^ inspired by 3D‐printed architectures found in the literature, accordingly.

Evaluating the suitability of previously mentioned methods (i.e., PµSL, TPL, µ‐SLS, and DIW) to bridge toward the micro‐optics manufacturing industry implies assessing their fabrication resolution capabilities denoted by the reachable feature size, which indicates the minimum reproducible feature size printable. **Figure** **1** shows a schematic representation of the feature size resolution range of PµSL, TPL, µ‐SLS, and DIW, and some illustrative optical devices inspired by 3D‐printed architectures are displayed. When looking across length scales (Figure 1), TPL is well‐known for possessing an ultra‐high resolution, achieving feature sizes up to and below 100 nm.^[^ ^49^, ^50^ ^]^ PµSL can print features down to 600 nm,^[^ ^51^ ^]^ but on average, 1–5 µm feature sizes are reported.^[^ ^52^ ^]^ PµSL reveals a remarkable improvement compared to its predecessor technologies, DLP and SL, with resolutions of 7.6 µm.^[^ ^53^, ^54^ ^]^ In addition, SL lately has been reported to stretch its capabilities up to 10 µm.^[^ ^55^, ^56^ ^]^ In contrast to VAT polymerization‐based methods, µ‐SLS reaches high resolution ranges between 5 to 50 µm,^[^ ^57^ ^]^ while standard SLS has reported ranges between 50 to 120 µm.^[^ ^34^ ^]^ On the other hand, the DIW counterpart has reports with possible feature size resolution at 600 nm with laser assistance,^[^ ^58^ ^]^ but typically, the literature suggests a range between 10 to 50 µm.^[^ ^36^, ^59^, ^60^, ^61^, ^62^ ^]^ A concise summary highlighting their central advantages and limitations and the resolution range achieved for different materials is shown in **Table** **1**. It should be noted that metals might not be suitable as materials for micro‐optics; thus, metals are only used in Table 1 to compare with other printable materials.

**Table 1:** Concise summary comparison of micro‐scale specific AM technologies: TPL, PµSl, DIW, and µ‐SLS.

Technology	Advantages	Technological limitations	Feature size resolution	Target material
TPL	Maskless direct writing approach permitting the fabrication of intricate, freeform architectures with ultrahigh precision	Relatively low polymerization scan speeds (≈20 mm^−1^s), and small printing area (10 × 10 µm to 2.2 × 2.2 mm)	91 nm^[^ ^50^ ^]^	Polymer
			350 nm^[^ ^74^ ^]^	Composite
			250 nm^[^ ^75^ ^]^	Ceramic
			200 nm^[^ ^76^ ^]^	Glass
			100 nm^[^ ^77^ ^]^	Metal
Projection Micro‐stereolithography	Combines relatively fast printing speeds for high‐resolution prints. Complete layer patterned with a single exposure	Light projection uniformity, layer‐by‐layer approach, and trade‐off between resolution versus printing speed	600 nm^[^ ^51^ ^]^	Polymer
			13.5 µm^[^ ^78^ ^]^	Composite
			12 µm^[^ ^79^ ^]^	Ceramic
			20 µm^[^ ^80^ ^]^	Glass
			25 µm^[^ ^81^ ^]^	Metal
Direct Ink Writing	Suitable for mass production due to minimum material waste, material‐properties tunability, and multi‐material compatibility	Poor vertical resolution, layer adhesion, and difficulty in achieving intricate architectures	1 µm^[^ ^82^ ^]^	Polymer
			14 µm^[^ ^61^ ^]^	Composite
			100 µm^[^ ^83^ ^]^	Ceramic
			100 µm^[^ ^84^ ^]^	Glass
			600 nm^[^ ^58^ ^]^	Metal
Micro‐SLS	No support structure is needed for intricate designs; high‐density materials are achieved	High surface roughness (5–25 µm), multi‐material limitations, and limited resolution	130 µm^[^ ^85^ ^]^	Polymer
			150 µm^[^ ^86^ ^]^	Composite
			50 µm^[^ ^87^ ^]^	Ceramic
			30 µm^[^ ^88^ ^]^	Glass
			5 µm^[^ ^35^ ^]^	Metal

It is worth noting that despite the technological advancements of these methods, their feature size resolution intrinsically depends on several factors, such as precursors used (i.e., photocurable resin, powder, or ink jetting), energy source or nozzle size, target printed material (i.e., polymer, composite, ceramic, or glass) and optical setup, which unavoidably translates into broad resolution ranges. However, it should be noted that in Figure 1, we highlight the inherent challenge of achieving 3D freeform optic elements when surpassing the micron mark feature size. For instance, this is the case of Kinoform lenses, which can focus up to 100% of incoming X‐rays and traditionally require multi‐step manufacturing strategies.^[^ ^63^ ^]^ Yet, the discussion of these millimeter‐sized optical devices can be found elsewhere^[^ ^64^, ^65^, ^66^ ^]^ and is out of the scope of this review.

Overall, Figure 1 and Table 1 show the concise summary comparison of micro‐scale specific AM technologies: TPL, PµSl, DIW, and µ‐SLS. Table 1 demonstrates the potential of TPL, which is capable of achieving submicrometer resolution over a comprehensive range of target materials. This offers the possibility of fulfilling one of the central ambitions of the optics field,^[^ ^9^ ^]^ i.e., to enable manufacturing capabilities to fabricate optical microelements with nanoscale precision. In terms of resolution, this is the case of Juodkazis et al., who explore TPL printing using SU‐8 photoresist, demonstrating the fabrication of mechanically stable free‐standing nanorods with lateral dimensions of ≈30 nm.^[^ ^72^ ^]^ The results from the literature show that TPL not only permits fabrication control on a multiscale level (i.e., micron‐sized freeform structures with feature sizes down to the nanoscale), but also presents the opportunity to investigate light‐matter interactions with unprecedented and limitless degrees of freedom not reachable by typical layer‐to‐layer AM processes. Thus, enabling 3D printing of custom microlenses (Figure 1).^[^ ^73^ ^]^

Another aspect that warrants further exploration is enlarging the library of compatible materials precisely designed for TPL. Until now, several interdisciplinary field efforts,^[^ ^21^, ^22^, ^23^ ^]^ including chemistry, physics, and materials science, have focused on tailor‐made photoresins formulations to enable micro‐printable functional materials (e.g., composites, metals, and ceramics/glass). We therefore intend to briefly assess the state‐of‐the‐art and relevance of polymers, composites, glass, and ceramics to bridge micro‐optics. Subsequently, we focus on the formulation chemistry behind GLACEs, which we foresee as upcoming micro‐optic materials. In addition, we reviewed the reported GLACEs that are compatible with TPL in depth. Last, we discuss how the photoresin formulation links to tuning optical properties by defect engineering in the target material and how this can unlock advanced micro‐optical systems.

## 3 Materials Suitable for TPL Micro‐Optics

TPL relies on photocurable resins that undergo cross‐linking upon light irradiation. More specifically, polymerization occurs by free radicals induced by TPA in photoinitiator (PI) molecules embedded in the resin, which promote the cross‐linking of surrounding monomers/oligomers.^[^ ^89^, ^90^ ^]^ PI molecules are employed due to their known TPA efficiency, assessed by the two‐photon cross‐section (σ_TPA_).^[^ ^91^ ^]^ In brief, σ_TPA_ denotes the probability that a molecule will absorb two photons simultaneously. The larger the σ_TPA_, the higher the TPA probability and thus the PI's excitation efficiency. In the literature, an extensive diversity of photoinitiator options exists typically under two categories: radical generation and H‐abstraction reaction, differing in the photochemical reactions stimulated under irradiation.^[^ ^92^, ^93^, ^94^ ^]^ PI molecules fall under different chemical families based on coumarins, chromones, flavones, and benzophenones, among other PI systems found elsewhere.^[^ ^95^ ^]^ According to A. Jaiswal and co‐authors, the most popular choices typically include ethyl‐2,4,6‐trimethylbenzoylphenylphosphinate, Irgacure 819, diazo‐naphthoquinone, benzil, isopropyl thioxanthone, 7‐diethylamino‐3‐thenoylcoumarin, and 4,4′‐ bis(diethylamino)benzophenone.^[^ ^49^ ^]^

Although the PI selection can have an effect on the TPL fabrication efficiency, other relevant contributing factors also play a role, e.g., the interaction between the PI and the formulation constituents and process parameters is unavoidable.^[^ ^89^, ^95^ ^]^ Thus, it is required to test the compatibility of monomeric species with the PI's reaction mechanism and conduct laser processing experiments (e.g., varying laser intensities and writing speeds) to obtain an optimal TPL fabrication. In some cases, the literature also sustains the employment of complementary photosensitizers, like plasmonic nanoparticles or semiconductor quantum dots, to improve the operability of PIs at trade‐off conditions (i.e., short fabrication window or slow polymerization rate).^[^ ^95^ ^]^ It should be noted that another approach is PI‐free photoresin. This is an important alternative as, in some cases, PI might be toxic in some settings. M. Malinauskas and co‐workers investigated 3D micro/nano‐fabrication at near dielectric breakdown irradiance, highlighting the significant role of avalanche absorption in free electron generation and chemical bond breaking. The work reveals that the photo‐initiation and chemical bond‐breaking steps in polymerization propagation differ from traditional multi‐photon absorption processes. The research provides insights into alternative TPL fabrication, which could be relevant for developing PI‐free printing approaches for GLACEs.^[^ ^96^ ^]^

Recapitulating, the core constituents describing the formulation of a TPL photoresin include a mixture of monomer‐based or oligomer‐based compounds, a two‐photon PI, and solvent(s). Moreover, the photoresin can integrate an inorganic‐rich payload to synthesize polymer‐derived materials (i.e., composites, ceramics, glass, etc.),^[^ ^23^ ^]^ which is discussed in the upcoming subsections. It is worth mentioning that detailed studies investigating diverse PI candidates and monomers in polymer‐derived materials have not been found in the literature. Last, some extra considerations for achieving an optimal photocurable resin^[^ ^22^ ^]^ are: i) having full optical transparency among the constituents within the utilized laser wavelength to maximize the σ_TPA_ of the PI employed. ii) Select the most suitable solvent(s) to solubilize the monomeric compounds and enhance the dispersion stability of inorganic precursors. iii) adjusting the laser power to surpass the threshold intensity required for polymerization and avoiding the ablation threshold of the polymerized 3D‐printed architectures will lead to higher spatial resolution and prevent overexposure damage.

### 3.1 Polymers

Since the invention of TPL, the principal material yielded during the fabrication of 3D architectures remains a fully organic polymer. In its early stages, the technological development of TPL suffered from the scarce availability of polymerizable photoresists, which were not optimized for TPA but for standard photolithography applications (working with SPA).^[^ ^97^ ^]^ An example includes one of the most prevalent photoresists employed in TPL, SU‐8, whose name originates from possessing eight epoxy groups. This material is the only epoxy‐based polymer utilized in TPL and popularly utilized in UV lithography. However, in recent years, dedicated research has been implemented to develop TPL‐specific polymerizable resins that efficiently operate with free radicals transferred from the PI.^[^ ^94^, ^98^ ^]^ These polymerizable resins fall into acrylate‐based monomers/oligomers.^[^ ^99^ ^]^

Acrylate‐based monomers have been utilized since the invention of TPL and still predominate as the most popular material chosen for printing, possessing a substantial variety of available functionalized monomers/oligomers.^[^ ^100^ ^]^ Commercially available acrylates typically include polymethylmethacrylate (PMMA), dipentaerythritol penta‐/hexa‐acrylate (DPPHA), pentaerythritol triacrylate (PETIA), pentaerythritol tetraacrylate (PETA), tris (2‐hydroxy ethyl) isocyanurate triacrylate, and SCR500.^[^ ^49^ ^]^ Moreover, companies like Nanoscribe GmbH have developed innovative formulations for rapid prototyping, leading to the IP‐series. These mostly predominant acrylate resins directly satisfy the fabrication of micro‐optical devices, offering properties tunability like high refractive index (RI) (≤1.62) and shape accuracy (i.e., high aspect ratio and low shrinkage). Explicit prospective applications, like 3D refractive optics, integrated photonics, and DEOs, can be explored in this direction.^[^ ^101^ ^]^ Furthermore, even biophotonic applications are available for some of the IP resins.^[^ ^22^, ^102^, ^103^, ^104^ ^]^

### 3.2 Composites

Deviating from fully organic polymer resins, a leading research scope within TPL focuses on developing hybrid polymers or composites to create advanced functional materials. In general, TPL composites utilize the polymer backbone as a matrix material while incorporating reinforcement components like inorganic monomeric salts^[^ ^105^ ^]^ (e.g., metal‐organic acrylates), inorganic non‐monomeric salts^[^ ^106^ ^]^ (e.g., metal‐organic alkoxides, nitrates, acetates, or chlorides), nanoparticles (NPs),^[^ ^107^ ^]^ nanotubes,^[^ ^108^ ^]^ or quantum dots.^[^ ^109^ ^]^ This inorganic payload can integrate additional properties, including piezoelectricity and stimuli‐responsivity. In particular, Arun Jaiswal and his collaborators^[^ ^110^ ^]^ studied the role of nitrogen‐doped carbon quantum dots as composite additives to create fluorescence‐encoded patterned nanostructures intended for anti‐counterfeiting tag purposes. These additives permit the TPL‐printed composite to reveal a hidden message tag that is only visible upon UV illumination. As for 4D materials^[^ ^111^ ^]^ (i.e., stimuli‐responsive systems), an example includes shape memory structures (SMS), which can respond to stimuli to shift their nanostructured coloration. For instance, Wang Zhang et al.^[^ ^112^ ^]^ reported submicron SMS where their size is tunable by heat/mechanical stress to achieve multi‐colored and even transparent states.

Finally, it is worth mentioning that composites are also attractive as precursors for polymer‐derived ceramics and glass when a thermal treatment such as pyrolysis or annealing is used. These tailor‐made hybrid photopolymers or composite precursor resins are currently available for manufacturing advanced micro‐optical devices. M. Malinauskas et al.^[^ ^113^ ^]^ conducted an extensive study to fabricate a series of micro‐optical elements, namely convex and Fresnel lenses, gratings, and solid immersion lenses using a zirconium‐siliconcomposite photoresin (SZ2080, IESL‐FORTH).

### 3.3 Glass and Ceramics

Given the infancy of 3D GLACEs, it is essential to recapitulate on prior fundamental concepts. Glasses are described as supercooled liquids that are thermally quenched to prevent crystallization.^[^ ^114^ ^]^ In other words, when transitioning fast from a liquid phase to a solid, the liquid undergoes a vitreous solid state, possessing an amorphous liquid‐like atomic arrangement. For this reason, we can induce glass transitions in other materials like polymers. However, due to the focus on micro‐optics, we only contemplate glass, which is highly applicable in optics.^[^ ^66^ ^]^ In addition to glass, ceramics have been widely standardized as non‐metallic inorganic solids, including oxides, nitrides, silicates, and carbides, among other advanced ceramics (e.g., SiCN, SiCO, SiBCN, SiAlCN).^[^ ^115^ ^]^ Within non‐metallic inorganic solids, we can subclassify them into crystalline, noncrystalline (i.e., amorphous), and polycrystalline ceramics.

Implementing a calcination or thermal annealing step, the decomposition of the organic polymeric backbone leads to miniaturized GLACE replicas. More specifically, tuning the atmosphere reactivity (e.g., ammonia and hydrogen sulfide) and the cooling rate influences the formation of glass or ceramic solid phases.^[^ ^116^, ^117^ ^]^ Concrete examples of reported GLACEs toward micro‐optical applications include TiO_2_^[^ ^118^ ^]^ and ZrO_2_.^[^ ^119^ ^]^ In the former publications, the authors reported TPL‐printable, fully‐dense TiO_2_ microarchitectures, demonstrating an RI of 2.3 and confirming a simulated photonic band gap experimentally by a woodpile geometry. As for the ZrO_2_ example, we reported the fabrication of 3D‐printed phosphors emitting in red, green, blue, and white by selectively doping ZrO_2_ microarchitectures with lanthanide species. Although other examples are found, polymer‐derived GLACEs achieved by TPL, the common ground is that TPL GLACEs lack a comprehensive understanding of the thermodynamic equilibrium induced within the 3D‐printed architectures. Therefore, future efforts on investigating the role of constituents within the formulation (contamination or defect sources), geometry design (altering the thermal flux within the microarchitecture), annealing conditions (target temperature, heating, and cooling ramp), and atmosphere pressure and reactivity (inert, oxidative, reductive) in the crystallographic phase formation, is essential to extend the library of available materials.^[^ ^24^ ^]^

### 3.4 Extending the Library of GLACEs for Micro‐Optics

Evaluating the suitability of GLACEs to bridge the micro‐optics industry implies understanding the concise needs within the field concerning technological characteristics. Achieving a submicron resolution to construct freeform micro‐optics is essential since intricate geometries such as the Kinoform X‐ray lenses (Figure 1) can be obtained. Another technological characteristic is having material‐choice‐flexibility in terms of optical properties, namely tunable RI and optical transparency/absorption at the relevant wavelength (λ) range, to operate in a wide range from UV to far infrared conditions. More specifically, materials with diverse RI, namely from negative refraction in metamaterials (N‐RI, n<0) to high‐refractive‐index alternatives (n>2), are fundamental in micro‐optics for correcting aberrations and achieving specific optical functionalities in micro/nano‐architected optical systems.^[^ ^120^ ^]^ Furthermore, achieving a homogeneous microarchitecture (i.e., yielding a single target‐crystalline/amorphous phase) is critical to avoid RI variations and scattering.

The surface roughness can cause pronounced scattering (typically denoted as Rsq). Thus, ultra‐smooth printed features (i.e., Rsq << relevant λ) are required even in curved surfaces to prevent surface scattering. Another compulsory characteristic includes long‐term stability (e.g., withstanding variable thermal, chemical, or mechanical conditions) to guarantee optimal performance at the industry standards. Finally, low‐temperature fabrication conditions are typically preferred to comply with complementary metal‐oxide semiconductor (CMOS) ‐related processes or preprocessing steps. **Table** **2** summarizes the overall fabrication functionalities and the most critical optical‐grade properties required in the field, along with an examination of whether these have been accomplished to date by the reviewed materials or not. It is important to note that polymers, composites, and metals are included to provide a general overview and be used for comparison with GLACE requirements.

**Table 2:** Ultimate characteristics required for micro‐optics regarding fabrication functionalities and optical‐grade material properties. Target material initials: polymers (P), composites (C), metals (M), and GLACE (G).

Ultimate characteristics	Criterion	Have been reached to date?	Refs.
a) Submicron resolution	Below 1 µm	Yes. TPL can reach subwavelength feature sizes at all the reviewed materials (i.e., P, C, M, and G); see Table 1 for reference.	[49, 50]
b) Tunable RI	From N‐IR (n<0) to high‐IR (n>2)	No. Mostly materials with RI below 1.62 (P and C). High RI and N‐IR alternatives are scarcely reported (M or G).	[23, 118, 121]
c) Tunable optical transparency/absorption	Above 90% at the relevant λ	Yes. Optically transparent materials (P, C, and G) or fully opaque (M) can be obtained.	[101, 122]
d) High bulk homogeneity	Above 99%	Yes. High homogeneity can be obtained for P, C, M, and G.	[3]
e) Optical‐grade surface roughness	Rsq << relevant λ	Yes/Partly. Reported values down to 3 nm (P and C). Reported values down to 5 nm for G. M require optimization.	[23, 123, 124]
f) Mechanical resilience	Quantitative	Yes. Various mechanical tests. However, standardization is required. See Table 3 for GLACEs.	[125]
g) Thermal and chemical stability	Quantitative /qualitative	No. Insufficient metrology to assess chemical and structural characteristics within the mesoscale.	[126]
h) Low‐temperature fabrication conditions	Below 600 °C	Yes/Partly. Polymers and composites can be fabricated at room temperature. Metals and GLACEs typically require temperatures above 500 °C.	[22]

Although polymers may satisfy the fabrication requirements (depicted as characteristics “a, c‐f and h” in Table 2) due to their advantageous direct processing bypassing post‐treatments, tuning their optical properties like RI (n>2) and improving their long‐term stability presents an undeniable limitation.^[^ ^23^, ^66^ ^]^ As for composites, some of these restrictions may be surpassed due to their enriching additives. Yet, the polymeric matrix imposes a trade‐off between which properties could be strengthened and which others cannot. Moreover, unsolved drawbacks have been reported, such as the lack of diverse matrix polymers, the impossibility of incrementing the additive loading due to undermining the printing resolution, and unfulfilled expectations about long‐term optical performance.^[^ ^55^ ^]^ Hence, the most prominent asset of these hybrid polymer composites relies on their precursor ability to yield printable materials (Table 2). Although not discussed, printed metals are frequently not implemented as primary optical‐active functional components in integrated devices. They are valuable due to the surface‐light interactions (plasmonics and metasurfaces) but could be addressed by supplementary deposition methods.^[^ ^127^ ^]^

TPL‐printable GLACEs are considered as the future trend in research, because they unlock 3D optical‐grade fabrication capabilities at the submicron scale, which is not feasible with other AM methods.^[^ ^128^ ^]^ GLACEs also possess mechanical, temperature, chemical, and radiation resilience, delivering the ultimate characteristics needed to catapult micro‐optic applications (Tables 2 and **3**).^[^ ^129^, ^130^ ^]^ It is worth noting that most of the discussion in the literature does not include measurements like optical transparency/absorption, surface roughness, and chemical/thermal resilience due to insufficient metrology advancements. This impact is emphasized with mechanical resilience measurements, where only a few citations reported this information (Table 3). Concerning optical performance, GLACEs provide a decent catalog of potential candidates to satisfy optical‐quality transparency and application‐driven RI,^[^ ^131^ ^]^ where high‐RI (n>2) materials are experimentally tested (Table 3).^[^ ^118^, ^132^ ^]^

**Table 3:** Reported characteristics of TPL‐printable GLACEs according to micro‐optics standards. Empty spaces imply unreported values at the given citations. Used abbreviations: Yttria‐stabilized Zirconia (YSZ, Y_2_O_3_:ZrO_2_), compression strength (CS, i.e., maximum stress recorded), ductility (D), Young's modulus (E), compressive strain to failure (CSF), stiffness (S), and electromechanical response (EMR).

Characteristic	Resolution [nm]	RI	Reported mechanical properties	Annealing temperature [°C]	Refs.
GLACEs					
BaZrO_3_	800	–	–	1000	[133]
CaZrO_3_	800	–	–	1000	[133]
Cr_2_O_3_	391	–	–	500	[25]
Glassy carbon	300	–	–	900	[134]
MnO_2_	391	–	–	450	[25]
SiO_2_	97‐170	–	CS and E	650	[124, 135]
SiOC	450	–	–	1000	[136]
SiCN	210	–	–	600	[137]
SiO_2_‐ZrO_2_	100	–	–	1000–1500	[130]
SiO_2_‐TiO_2_	200	–	–	600–1000	[138]
SnO_2_	150	–	–	500	[139]
SrZrO_3_	800	–	–	1000	[133]
TiO_2_	300	2.3	–	900	[118]
YSZ	410	–	CS and D	600–1200	[140]
ZnO	250	–	CSF, S, and EMR	500	[75]
ZrO_2_	200	2.1	CS, D, E, and CSF	600–1200	[132, 141]

As for the annealing temperatures, some candidates comply with the low‐temperature fabrication range (below 600 °C), while others require elevated temperatures (up to 1500 °C), see Table 3. Furthermore, the impressive fact of micro/nanostructured ZrO_2_^[^ ^132^ ^]^ and ZnO^[^ ^75^ ^]^ reporting compressive strain to failure results, competing with their macroscopic counterparts. These results invites us to explore future characterization approaches and gain a deeper understanding of material properties.

Overall, limited GLACEs have successfully been 3D printed by TPL, and further efforts are needed to bring macroscale GLACEs into the nanoscale realm. According to the material characteristics and optical properties of other reported bulk alternatives (e.g., Al_2_O_3_, MgO, and MnO), GLACEs succeed in the field standards.^[^ ^25^, ^131^ ^]^ As suggested by Diana Gonzalez‐Hernandez and collaborators,^[^ ^126^ ^]^ progress urges the scientific AM community to characterize 3D microarchitectures in detail. Not only to qualify GLACEs according to optical‐grade specifications, but also to standardize a protocol focusing on reproducibility and repeatability. Yet, we envision future research perspectives targeting GLACEs as trending materials for manufacturing 3D micro‐optics. For this reason, we examine the state‐of‐the‐art about GLACEs hybrid photoresin formulation below.

## 4 Pre‐GLACE Chemistry

**Figure 2:**
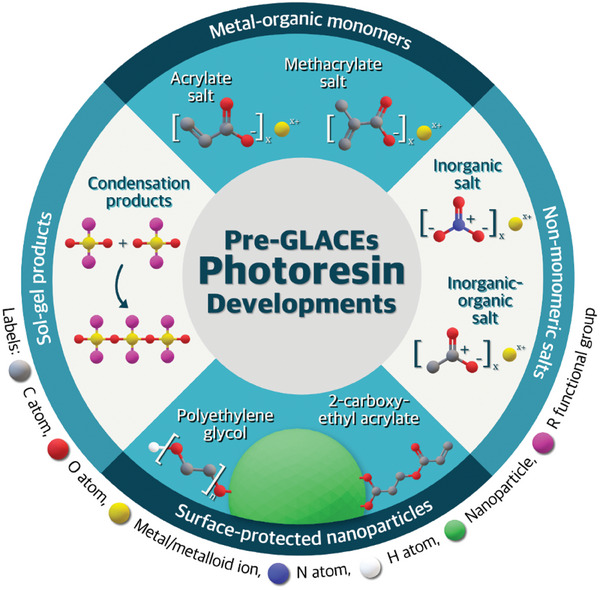
Overview of the photoresin developments for pre‐GLACEs. Schematic labels: carbon atoms (grey spheres), oxygen atoms (red spheres), metal or metalloid ions (yellow spheres), nitrogen atoms (blue spheres), hydrogen atoms (white spheres), surface‐protected nanoparticles (green spheres), and functional group R (pink spheres) –, e.g., H, CH_3_, C_2_H_5_. Substitute R with R’ –, e.g., acrylates or di‐epoxy compounds for monomers.

As discussed in the section 3. 3, TPL photoresin formulations yielding GLACE replicas (pre‐GLACE) can be chemically tuned. **Figure** **2** shows the most studied photoresin developments for pre‐GLACEs consisting of four inorganic loading routes, namely (i) metal‐organic monomeric salts, (ii) non‐monomeric salts (i.e., fully inorganic salts like nitrates or inorganic‐organic salts like acetates), (iii) surface‐protected NPs, and (iv) sol‐gel products. The presented routes are primarily organic‐based, but water‐soluble photoresins should be considered to reduce the imposed environmental pressure over organic solvents used in acrylate photoresins.^[^ ^25^ ^]^

Among the various pre‐GLACE photoresin synthesis routes, metal‐organic monomeric salts have been a popular option, particularly pre‐GLACE for low‐temperature post‐processes.^[^ ^142^ ^]^ Metal‐organic monomers are complex salts of metal ions coordinated with organic‐derived ligands, like acrylates. As shown in Figure 2, depending on the valence of the metal cation (superscript x), the number of acrylate or methacrylate ligands will coordinate. The core advantage of these metal‐organic monomers relies on their utilization as cross‐linking coagents, sharing identical monomer groups with the polymer resin employed. In this manner, the inorganic loading is cross‐linked with the parental acrylate backbone, improving its dispersion and stability. Examples found in the literature include our previous work on alkaline‐earth perovskites (i.e., BaZrO_3_, CaZrO_3_, and SrZrO_3_),^[^ ^133^ ^]^ where the stoichiometry‐precise formulation of alkaline‐earth acrylates with zirconium acrylate is critically needed to yield the target perovskite crystalline phase. For CaZrO_3_, it has been found that Ca‐excess (beyond the equimolar ratio used with Ba and Sr counterparts) is needed to overcome undesired secondary phases after annealing. Although other examples originate in the literature, it is worth mentioning that most metal‐organic acrylates are currently discontinued by commercial suppliers, leading researchers to opt for tailor‐made compounds with ligand‐exchange reactions.^[^ ^56^ ^]^

Non‐monomeric salts are standard metallic compounds composed of metal cations coupled with inorganic anions such as nitrates or organic anions like acetates, as depicted in Figure 2. These non‐monomeric salts are diluted in the resin formulation, intending to reinforce the polymeric backbone as non‐bonded or dispersed additives. In other words, the inorganic loading is not bonded to the polymer chains due to the lack of compatible functional groups. As a reference, Yee and collaborators^[^ ^75^ ^]^ reported TPL‐fabricated ZnO microarchitectures by preparing a custom‐made resin composed of zinc nitrate hexahydrate. The authors maintained a high Zn concentration (51 wt.%) without affecting the resin homogeneity and long‐term stability. This route offers a more extensive catalog of non‐monomeric inorganic salts for printing GLACEs; however, the choice of this route also accounts for formulation limitations, including solubility and dispersion stability with the solvents and monomers employed and leaching out of the non‐bonded metal ions after developing the 3D‐printed structure. In addition, with higher weight fraction loadings, bulk defects on the 3D‐printed polymer structure can be promoted, such as agglomerates, fractures, or increased brittleness.

Alternatively, using surface‐protected NPs as inorganic loading is a typical approach for TPL‐printable GLACEs, where the stoichiometry and solubility factors are no longer relevant for higher‐weight fraction loadings. Still, surface protection is required in the NPs to prevent oxidation and improve their homogeneous dispersion and stability within the liquid resin (i.e., diminishing their aggregation and agglomeration). The characteristics facilitate their integration in the cross‐linked polymer with monomer‐compatible functionalization. As shown in Figure 2, the most common surface‐functionalization prospects employed in the literature are polyethylene glycol (PEG) and 2‐carboxy‐ethyl acrylate. For example, Wen and co‐authors^[^ ^135^ ^]^ reported TPL‐printable silica nanostructures using PEG‐functionalized colloidal silica NPs. To achieve sub‐200 nm resolution during printing, the authors declared that NPs must be small enough (≈10 nm), highly translucid (i.e., matching the RI of the photoresin), and possess high thermal conductivity to prevent localized vaporization. In this manner, energy deposition variations in TPL due to RI differences and thermal conduction are suppressed. These variations can lead to voxel size inconsistencies and overexposure damage in the photoresin.^[^ ^24^ ^]^

Last, sol‐gel (Figure 2) is another predominant approach for pre‐GLACE formulation due to its well‐established foundations in GLACE synthesis in several application fields, from optics and photonics to energy storage, catalysis, and surface modification. The sol‐gel method in TPL, compared to the standard photoresin formulation of the other mentioned approaches, requires two extra preparation steps. First, the inorganic loading (i.e., metal alkoxides), combined with monomers or oligomers, solvents, and TPI, is incorporated with water to hydrolyze and condense into a colloidal suspension solution (known as sol). Second, the sol is heated at low temperature to undergo gelation by removal of solvents. Afterward, the prepared sol‐gel is ready for TPL photopolymerization, where the TPI induces the cross‐linking of the sol‐gel polymer structure. Examples of this route include the work of Gailevičius et al.^[^ ^130^ ^]^ which investigates the fabrication of a hybrid GLACE (i.e., possessing SiO_2_ and ZrO_2_. This sol‐gel formulation consisted of methacryloxypropyl trimethoxysilane and zirconium propoxide.^[^ ^143^ ^]^ The advantage of this SiO_2_ and ZrO_2_ sol‐gel formulation is that it skips the usage of a TPI during printing. The approach does not compromise resolution, surface roughness, and structural fidelity.

## 5 From Pre‐GLACEs to GLACE Architectures

**Figure 3:**
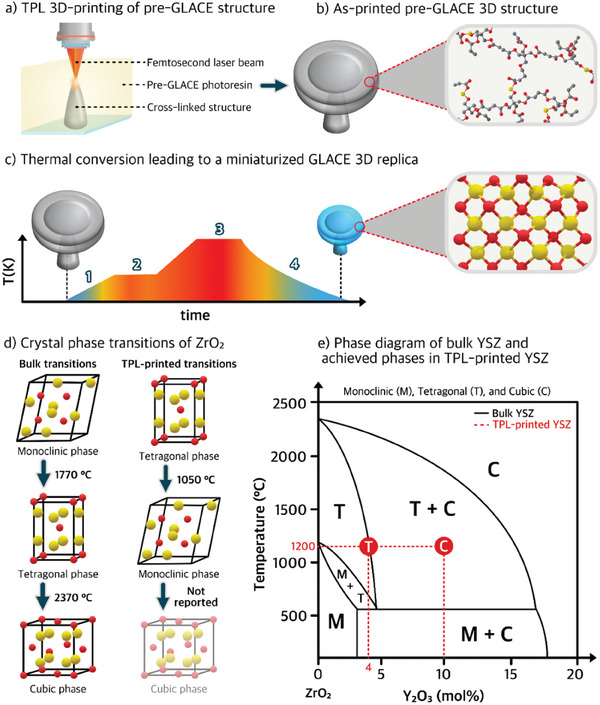
Schematic representation of the thermal conversion leading to a miniaturized GLACE replica, representing the a) printing step of a pre‐GLACE microarchitecture via TPL, b) as‐printed pre‐GLACE 3D microarchitecture showing the cross‐linked chemical nature with inorganic bonded additives, and c) miniaturized GLACE conversion after thermal/annealing exposure, highlighting the inorganic chemical network exemplified as a binary metal oxide. Exemplary d) crystal phase transitions of ZrO_2_ in bulk and TPL‐printed microarchitectures, and e) phase diagram of bulk YSZ, highlighting (in red dashed‐line) equiv. stabilization for tetragonal zirconia at 4 mol% Y_2_O_3_ and low‐temperature phase stabilization for cubic zirconia at 10 mol% Y_2_O_3_ in TPL‐printed microarchitectures.^[^ ^140^ ^]^

Once the photoresin is ready, printing the pre‐GLACE (**Figure** **3**a) is the next step. The pre‐GLACE is subjected to high temperatures to derive the GLACE replica. The combustion conditions can vary depending on the pre‐GLACE loading route, where, e.g., the metal additives can be backbone cross‐linked pre‐GLACEs (Figure 3b).^[^ ^144^ ^]^ Understanding the temperature range at which most organic matter decomposes and metal additives nucleate into nanocrystal grains is necessary. N. Yang and K. Lu^[^ ^145^ ^]^ conducted a comprehensive study investigating the thermodynamic equilibrium in polymer‐derived bulk GLACEs, yielding silicon oxycarbide (SiOC) co‐doped with a transition metal “M” (M = Ni, Mo, Co, and Zr) at a 1:0.02 ratio. In a combined effort, experimentally and computationally, the authors constructed a quaternary composition diagram for each SiOC/M system, revealing insights into phase separation pathways for the transition metals (i.e., pure metal, metal carbide, metal silicide, and metal oxide). However, the authors remarked that their computational model is contemplating the thermodynamic equilibrium in an ideal state, bypassing the kinetic aspects of phase separation and crystallization. It can be speculated that phase formation and segregation are connected to how crystals grow, which depends on the pre‐GLACE chemical environments. To date, such studies are limited.^[^ ^133^ ^]^ It is, therefore, important to stress the significance of understanding the thermodynamic stability of crystallization for polymer‐based GLACEs during thermal treatment (Figure 3c–e).

As for TPL‐printable GLACEs, various studies have addressed the contributing factors toward amorphous/crystalline phase formation, as summarized in **Table** **4**. First, inorganic weight fraction loading. Several authors have identified a predominant linear isotropic shrinkage behavior relying on the inorganic loading weight percentage, implying that a lower shrinkage percentage will occur at highly loaded photoresins.^[^ ^75^, ^136^, ^146^, ^147^ ^]^ In this direction, an insufficient inorganic weight fraction occasions uncontrolled shrinkage, prone to obtaining an incomplete crystallization (amorphous phases) and losing geometrical fidelity.

**Table 4:** Reported factors contributing to the phase formation of GLACEs.

Reported factors	Contribution to GLACE conversion	Refs.
a) Inorganic weight fraction loading	Linear contribution toward GLACE isotropic shrinkage. Insufficient inorganic weight fraction loading can hinder crystallization and geometrical integrity.	[75, 136, 146, 147]
b) Photoresin's organic composition	Undesired secondary phases sourced from organic constituents can interfere with the GLACE conversion. On the other hand, the characteristics of the monomeric species (i.e., chain length and conformal flexibility) can promote GLACE conversion.	[79, 124, 141, 148]
c) Heating/ cooling rate	The heating rate allows for a controlled combustion of inorganics, permitting better tunability of the yielded GLACE. The cooling rate is critical when choosing between glass/amorphous states and highly crystalline phases. Still, an aggressive cooling can lead to bulk defects (e.g., cracks and voids).	[144, 150, 151]
d) Glace‐target temperature	The GLACE‐target temperature bifurcates into glassification or ceramization processes, allowing the selection of a desired material. Proper tuning and optimization are required when following bulk‐related phase formation conditions.	[24, 124, 150]
e) Dwelling time and pre‐dwelling time	The dwelling time is essential to obtain the GLACE target phase with the desired characteristics. Its tunability is necessary to meet optical‐grade standards. Also, implementing a pre‐dwelling time is suggested to promote organics removal at lower temperatures.	[144, 150, 152]
f) Atmosphere type/ pressure	The reactivity and pressure level of the atmosphere can influence the obtained composition, enhancing or suppressing reactions occurring during the combustion/calcination of organics.	[153, 154, 155]
g) Phase‐stabilizing dopants	Employing phase‐stabilizing impurities in the formulation can lower the phase‐formation temperature, facilitating the target GLACE conversion.	

Other studies have addressed the role of photoresin organic composition. For instance, due to the carbon‐rich nature of the pre‐GLACE structure, the formation of graphitic and disordered carbon impurities (i.e., buried within the GLACE microarchitecture) is reported in the literature,^[^ ^75^, ^141^, ^148^ ^]^ which agrees with the previously described thermodynamic studies for polymer‐derived bulk GLACEs.^[^ ^145^ ^]^ In these studies, it has been found that carbon‐derived impurities can interfere with the GLACE conversion by reacting undesirably with the metal additives (e.g., forming carbonates and affecting the stoichiometry) or even forming GLACE composites, improving the mechanical strength but compromising the aimed phase of the printed microarchitecture.^[^ ^141^ ^]^ Moreover, the cross‐linking degree of the printed pre‐GLACE structure is another factor that can impact the chemical bond rearrangement and phase transformation. Studies have revealed that the more ordered the cross‐linked network (i.e., depending on the monomer chain length and conformational flexibility), the easier the amorphous‐to‐crystalline transition.^[^ ^79^, ^124^, ^149^ ^]^

Another noticeable factor derives from the choice of solvents, where the implementation of halogen‐rich solvents (e.g., dichloromethane) or nitrogen/sulfur‐rich solvents (e.g., dimethylacetamide and dimethyl sulfoxide, respectively) can lead to undesired secondary phases. This example aligns with CaZrO_3_, which shows residual traces of CaSO_4_ sourcing from dimethyl sulfoxide.^[^ ^133^ ^]^ Although the last‐mentioned factors (i.e., photoresin's organic composition and inorganic weight fraction loading) play a role in achieving the target GLACE phase, the thermal conversion conditions are the primary factor contributing to the polymer‐to‐GLACE transition. Table 4 presents the contributing factors that can affect GLACE phase formation.

According to diverse reports in the literature,^[^ ^22^, ^24^, ^66^, ^124^, ^126^, ^150^ ^]^ tuning and optimizing the thermal treatment of the pre‐GLACE is paramount to achieving optical‐grade high‐quality GLACE replicas. The adjustable thermal conditions typically include (Table 4): (i) the heating/cooling rate, (ii) the glassification/ceramization target temperature (hereafter GLACE‐target temperature), (iii) the dwelling time, and (iv) the atmosphere type/pressure.

The initial step of GLACE conversion starts with a heating ramp, depicted as step 1 in Figure 3c. During this step, the heating rate must be low enough (e.g., 0.5‐1 °C/min) to promote favorable/not‐aggressive combustion of organic constituents.^[^ ^144^, ^151^ ^]^ Consequently, the emitted combustion gases can gradually be removed or released from the pre‐GLACE structure, avoiding undesired reactivity with the metal counterparts. It is worth adding that some reports^[^ ^141^, ^149^ ^]^ also suggest using a pre‐dwelling time (i.e., the period during which a material is held at a specific temperature before the target heating or treatment process begins) to ensure all the combustion products are released, as seen in step 2 of Figure 3c. In this manner, someone can hypothesize that the 3D‐printed structure could have sufficient time to gradually release the organic‐derived gases, yielding an organic‐free amorphous/glass phase, which can then be heated further for the desired glassification/ceramization evolution. Although the latest hypothesis might be correct, a proof‐of‐concept study is required.

The GLACE‐target temperature, visually depicted as the highest processing temperature in step 3 of Figure 3c, typically relies upon thermal‐induced phase formation temperatures of bulk counterparts. In other words, to select the desired phase's GLACE‐target temperature, one could follow reported annealing/calcination conditions for bulk materials. However, the readers should bear in mind that the pre‐GLACE components might lead to unconventional phase transitions, as in the case of ZrO_2_, which can lead to low‐temperature phase stabilization of the tetragonal phase (see Figure 3d).^[^ ^141^, ^156^ ^]^ Other studies enable the targeted phase, like TPL‐printed rutile nanocrystalline TiO_2_ annealed at 900 °C.^[^ ^118^ ^]^ TiO_2_ results are in line with both experimental and simulation reports in the literature to achieve predominately the rutile phase.^[^ ^157^, ^158^, ^159^ ^]^ It is worth remarking that glass phases naturally require lower processing conditions (i.e., ≈600 °C), while highly crystalline phases tend to employ higher temperatures (e.g., up to 1500 °C).^[^ ^24^ ^]^

Aligned with the GLACE‐target temperature, the dwelling time (i.e., the thermal treatment duration at a target temperature for the desired glassification/ceramization process) is essential to achieve the desired characteristics in the converted material, like grain size, enhancing predominant phase, release lattice stress, and densification.^[^ ^152^ ^]^ Although it may seem like more prolonged dwelling times result in high‐quality GLACEs, the proper tunability of dwelling time^[^ ^144^, ^150^ ^]^ is critical to meeting optical‐grade criteria (e.g., surface roughness and grain size). Still, we point out the importance of optimizing and conducting temperature influence studies due to the impact of the other factors playing a role in GLACE conversion. For example, TPL‐printed ZrO_2_^[^ ^156^ ^]^ shows tetragonal phase (t‐ZrO_2_) stabilization at 600 °C, while its monoclinic phase (m‐ZrO_2_) is at 1050 °C, exemplifying an atypical behavior compared with bulk zirconia phase transitions, where m‐ZrO_2_ is stable at room temperature and transitions to t‐ZrO_2_ above 1170 °C (see Figure 3d).^[^ ^160^, ^161^, ^162^ ^]^ Therefore, we see benefits in constructing phase diagrams for TPL GLACEs systems, as depicted in Figure 3e. The benefit of such dedicated work is that it can target specific optical characteristics necessary for specific optical functionality. Other factors, like prolonged annealing over crystalline materials, may not affect crystal grains or might tend to grow crystal grains very slowly. The latter could have a detrimental effect, leading to shape changes within the 3D GLACE architecture if the grain size becomes more prominent than the feature size.

Other factors that should be included in GLACE are the atmosphere reactivity (i.e., inert or reactive) and pressure level (i.e., vacuum, atmospheric pressure, or high pressure), which without doubt will alter the GLACE characteristics (Table 3). The variation in atmosphere type leads to different thermal treatment processes, where pyrolysis/calcination intends to avoid the combustion of the material by the absence of oxygen, and annealing uses oxygen‐rich (air) to promote it. According to combustion behavior studies of diverse organic polymers in bulk (e.g., polylactic acid, polycaprolactone, polybutylene succinate, among others)^[^ ^155^ ^]^ conducted in oxygen‐rich environments, the main decomposition products are CO_2_ and H_2_O. Furthermore, in oxygen‐free environments (i.e., N_2_), a mixture of oligomers, unsaturated carboxylic acids, CO_2_, CO, and H_2_O can be observed. These organic‐derived decomposition products can potentially react with the nucleating grains, forming oxides, carbonates, carbides, or even nitrides and sulfates when nitrogen and sulfur are present either in the environment or the photoresin.

The GLACEs typically use an oxygen‐rich/air atmosphere for metal oxide formation (i.e., TiO_2_, ZrO_2_, ZnO, SnO_2_, SiO_2_, YSZ, and alkaline earth perovskites), while an inert atmosphere using ultra‐pure nitrogen flow yields oxygen‐deficient GLACEs (i.e., SiOC, SiCN, and glassy carbon). As for the role of atmosphere pressure, scarce reports have investigated its impact on TPL‐printed GLACEs. However, in bulk polymer‐derived GLACEs, studies have reported that the pressure level (i.e., partial pressure of the gas component) has a strong effect in enabling or suppressing desired reactions.^[^ ^150^, ^153^ ^]^ For instance, thermal treatments in a vacuum promote carbothermal reduction reactions, which facilitate the removal of oxygen and gaseous decomposition products and reduce the metal additives (primarily applicable to yield metallic structures).^[^ ^77^ ^]^ On the contrary, pressurized environments bypass carbothermic reactions to permit metal additives to react with O_2_, N_2_, H_2_O, or CO/CO_2_.^[^ ^154^, ^163^, ^164^ ^]^

Last, the GLACE conversion mechanism concludes with the cooling‐down phase (see step 4 of Figure 3c), contributing to achieving glass or ceramic replicas. As previous sections hinted, glass/vitreous phases imply rapid cooling (thermal quenching). However, a rapid cooling rate leads to a glassy structure, which could be prone to bulk defects like cracks or voids/pores due to aggressive thermal shock.^[^ ^122^, ^165^ ^]^ To overcome this, utilizing sufficient low‐temperature conditions (≈650 °C) can yield an amorphous (glass‐like) phase to prevent crystallization.^[^ ^116^, ^130^ ^]^ Thus, to achieve high‐quality glass microarchitectures, a trade‐off between the glass target temperature and the cooling rate should be considered.

As for ceramics, reports in the literature mainly incline for a natural‐rate cooling step to permit a gradual relaxation of the achieved crystallographic phase.^[^ ^136^, ^137^, ^138^ ^]^ In this manner, the thermal stress is relieved, and the lattice can re‐arrange toward a higher crystallinity degree. A gentle cooling ramp (≈2 °C/min) can be utilized, but it requires further optimization and depends on the chosen target material. Homologous to glass, when ceramics are cooled too abruptly, the microarchitectures may collapse or contain bulk defects formation.

In short, the thermal conversion of GLACEs comprises several experimental factors considering both formulation and thermal conditions (Table 4) that, upon optimization and tuning, uphold the expected GLACE target phase. Still, it is worth remarking that new studies are investigating using phase‐stabilizing dopants to promote a specific GLACE phase at lower temperatures. For example, TPL‐printed YSZ with a doping concentration of 10 mol% (i.e., using yttrium monomeric salts) showed a cubic‐phase stabilization from 600 °C to 1200 °C^[^ ^140^ ^]^ (see Figure 3e), which is typically stable above 2370 °C at pristine conditions.^[^ ^160^, ^161^, ^162^ ^]^ Moreover, phase‐stabilizing dopants can also be used by using NPs, where reports of yttria‐stabilized ZrO_2_ NPs have also been conducted.^[^ ^132^ ^]^ We remark that exploiting defect chemistry, also known as defect engineering, is typically neglected by TPL‐based GLACEs compared to bulk counterparts. Therefore, given the already defective nature of polymer‐derived GLACEs^[^ ^24^ ^]^ and the pivotal role of defect chemistry in tunning the optical properties in optics.^[^ ^9^, ^166^, ^167^ ^]^ We examine the applicability of defect manipulation in TPL‐based GLACEs and, more specifically, ceramics exclusively. Hence, this defect assessment addresses fundamental and potential applications of unwelcome imperfections within the ceramics described below.

## 6 Intrinsic and Extrinsic Defects in Ceramics

Defects, both intrinsic and extrinsic, significantly influence the properties of ceramics. Intrinsic defects arise from defects in the ceramic, such as vacancies and interstitials, while extrinsic defects result from intentional impurities or additives.^[^ ^168^, ^169^ ^]^ These defects alter the arrangement of atoms, density, and various mechanical, thermal, electrical, and optical properties.^[^ ^170^, ^171^, ^172^ ^]^ In the context of AM, understanding and manipulating defects may be crucial for changing ceramic properties, particularly in micro‐optical functional devices.^[^ ^24^, ^140^, ^141^, ^173^ ^]^

A key aspect to understand when dealing with imperfections like defects is that defects are typically most prominent at the interface as AM of ceramics relies on uncontrolled crystal formation owed to thermal decomposition.^[^ ^174^, ^175^ ^]^ Such uncontrolled crystal formation in micro‐optical devices becomes increasingly important.^[^ ^173^ ^]^ In the following, we focus on why defects are beneficial for developing ceramic‐based micro‐optics. Furthermore, we discuss typical dopants (aliovalent, transition metals, and lanthanides) used in micro‐optics to produce functional materials.

**Figure 4:**
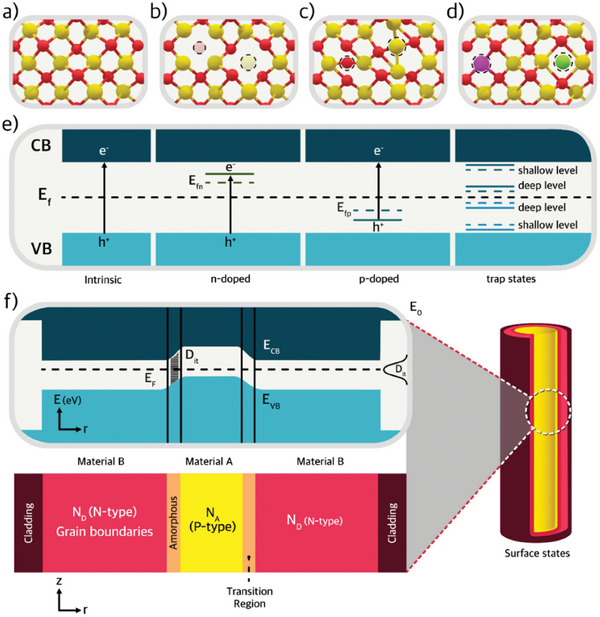
Schematic depiction of a) perfect lattice, b) atomic vacancies, c) atomic interstitials, d) substitutional and interstitial dopant atoms, e) band gap schematics, and f) 3D multi‐material arrangement including p‐ and n‐type semiconductor in thermal equilibrium including a description of possible defects encountered in such a configuration and the existence of surface states (D_it_). The vacuum energy (E_0_), conduction band energy (E_C_), valence band energy (E_V_), and Fermi energy level (E_F_) are indicated. An insulating cladding layer is also included.

First, we should identify the type of defects in ceramics, which can be intrinsic and extrinsic point defects.^[^ ^176^, ^177^, ^178^ ^]^ Intrinsic defects include vacancies, interstitials, and defect complexes.^[^ ^179^, ^180^ ^]^ An example of an ideal crystal and a vacant site, the latter being atoms missing within the lattice, is given in **Figure** **4**a,b. The interstitial point defect in a crystalline material is fundamentally different and is characterized by additional atoms that occupy positions between regular lattice sites, as presented in Figure 4c. Aside from intrinsic defects, extrinsic defects are intentional impurities or dopants introduced to the crystal. Examples are substitutional dopants and interstitial dopants. Substitutional dopants can replace atoms in the crystal lattice, as depicted in Figure 4d, while interstitial dopants occupy sites between lattice atoms, also shown in Figure 4d. A reflection on how the defects can be used to tune GLACEs can be highlighted when thinking about semiconductors. In the case of semiconducting ceramics, the presence of n‐type defects shifts the Fermi energy toward the conduction band, while p‐type defects move it toward the valence band,^[^ ^181^ ^]^ as depicted in Figure 4e.

From the semiconductor example, it is safe to say that defect engineering is a way toward p/n functionality.^[^ ^182^, ^183^ ^]^ In the realm of optics, intrinsic defects, for instance, influence light absorption and transmission, which is essential for controlling optical characteristics like light‐emitting or absorbing devices.^[^ ^184^, ^185^, ^186^ ^]^ Moreover, these defects can also modify the RI of ceramics,^[^ ^187^ ^]^ which is crucial for regulating light propagation.^[^ ^188^ ^]^ In a non‐ideal crystal, it is more common to encounter defect complexes rather than individual point defects.

Considering extrinsic defects, dopants are crucial in tailoring material optical and electronic properties to meet specific device requirements. Common dopants include aliovalent ions, transition metals, and lanthanides, each serving distinct purposes. Aliovalent dopants modify conductivity and RI by introducing charge carriers into the crystal lattice. Transition metals introduce energy levels within the bandgap, enabling precise tuning of electrical and optical properties. Lanthanide dopants offer unique optical characteristics suitable for high sensitivity, particularly thermometry.^[^ ^189^ ^]^ Furthermore, lanthanide offers further functionalities in laser technology due to its narrow spectral bandwidth.^[^ ^190^ ^]^

Strategic dopant selection and optimization are critical for customized optical performance. However, the intricate interplay with other defects can be equally important, especially in the case of microscopic confinements.^[^ ^191^, ^192^ ^]^ As the defect concentration rises, discernible energy states, called trap states, may emerge within the energy levels of the bandgap.^[^ ^181^, ^182^ ^]^ Unlike the Fermi energy level, trap states represent accessible energy levels within the bandgap. Trap states^[^ ^193^ ^]^ in semiconductors can be broadly categorized into shallow and deep states based on their proximity to the band edges,^[^ ^194^ ^]^ as shown in Figure 4e. Trap states can significantly impact carrier mobility and recombination rates directly related to the chemical potential, thus influencing the overall electrical behavior of the material. An additional scenario is an interface of dissimilar materials with differing electronic properties. Such an interface can create barriers to charge transport, affecting electronic device performance. Optically, when incident photons encounter trap states at interfaces, they can undergo scattering, leading to deviations in the direction of propagation. This scattering process contributes to the transmission light's loss of coherence and directionality, directly impacting transparency and reflectivity.

Now that key factors have been identified, a question remains open, i.e., how to circumvent undesired defects within crystalline microarchitectures. Some mitigation strategies, like rapid thermal annealing or annealing under various gas environments and pressures, might lead to circumventing undesired functionalities. Although such mitigation strategies aim to decrease defect concentration, one must also reckon with the thermodynamic minimum of the intended crystal structure. This consideration underscores the delicate balance between achieving the desired crystal structure and reducing defect concentration. This is underlined when one compares the ratio of the number density of dopant atoms to the number density of atoms in the bulk crystal to the ratio of the surface density of defects to the surface density of atoms on a crystal plane. Where in the bulk, the ratio is typically in the range of 10^−3^ to 10^−8^, the ratio can be as high as 10^−2^ at the surface. As the surface‐to‐volume ratio in architectures increases, the interface may be profoundly influenced by the density of defects.

Moving from a single element to an interface with dissimilar compositions, integrating multi‐material 3D printing (multi‐material integration within the same architectures or the use of different materials for distinct architectural features), especially with TPL, offers exciting prospects for creating complex architectures with tailored defects. An example is given in Figure 4f. Here, a heterojunction is presented as being part of a complex 3D microarchitecture. This type of TPL design remains unexplored but presents intriguing possibilities when combined with optical‐driven defects. Researchers can address inherent challenges in existing fabrication processes (Figure 4f) by adopting a multi‐material approach. When engineered appropriately, intrinsic and extrinsic defects can enhance device functionality, particularly within 3D AM, giving additional multi‐material patterning freedom. This cutting‐edge opportunity extrapolates to studying 3D‐printed multi‐compositions and positioning multiple extrinsic defects (i.e., doped photoresins) in space alongside undoped ceramics.

Although the spatial manipulation of vacancies and interstitials is unattainable only by different photoresins, an illustrative application is 3D printing intrinsic‐defective heterostructures such as Cu_2_O/ZnO, where the optoelectronic properties (e.g., energy band alignment and optical absorption) could be tuned in principle by copper vacancies in Cu_2_O and oxygen vacancies or dopants like Al in ZnO.^[^ ^194^, ^195^ ^]^ Obtaining 3D‐printed multi‐GLACE compositions not only allows for the spatial manipulation of defects, but also enables the study of defect gradients over an interface.

## 7 Multi‐Material Printing of GLACEs

Multi‐material AM has emerged to create complex structures by incorporating multiple compositions into a single architecture^[^ ^196^, ^197^ ^]^ or multi‐material printing of different materials in specific locations of the same 3D architecture (Figure 4f). Various applications can be found for multi‐material printing. Some include dielectrics, magnetic, active, catalytic, and living materials.^[^ ^198^, ^199^, ^200^ ^]^ For example, A. Saha et al. recently demonstrated the multi‐material printing capabilities by manufacturing catalytically active living materials using yeast‐laden hydrogel inks. The approach leverages the production of multi‐stimuli‐responsive materials within the bioengineering domain.^[^ ^201^ ^]^ In another work, Yu et al. theoretically showed that customized architectures with enhanced mechanical resilient characteristics can be produced by optimizing the architecture mesostructures.^[^ ^202^ ^]^ Yu et al. theoretical foundation can be used as a stepping stone for TPL, particularly within mesoscale GLACEs.

Printing mesoscale architectures with mixed micro‐ and nanometric dimensions within a defined volume has been demonstrated with TPL. However, spatial patterning of multiple compositions is not trivial; it can be done by integrating fluid management systems with TPL. F. Mayer and co‐authors^[^ ^22^ ^]^ recently implemented a dedicated TPL‐integrated microfluidic cell to exchange photoresins during printing. This led to the possibility of printing multiple compositions within the same microarchitecture without disrupting the single‐step manufacturing process. The authors suggest that the approach can be scaled up to allow the polymerization of multiple photoresins simultaneously.^[^ ^203^, ^204^, ^205^ ^]^

**Figure 5:**
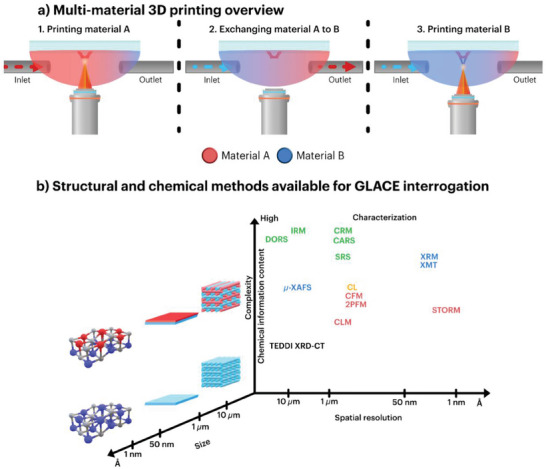
a) Multi‐material composition printing sketch. After printing and development, the precursor for glass/ceramic is annealed. b) Depending on the information needed and the spatial resolution at which the GLACE requires interrogation, a suitable characterization method can be selected to evaluate the chemical composition and crystallinity in space and time for single and multi‐material GLACEs. The abbreviations of the included characterization techniques are as follows. Vibrational spectroscopy methods (green): DORS, diagonally offset Raman spectroscopy; IRM, infrared microscopy; CRM, confocal Raman microscopy; CARS, coherent anti‐Stokes Raman spectroscopy; SRS, stimulated Raman scattering microscopy. Electronic spectroscopy methods (red): CFM, confocal fluorescence microscopy; 2PFM, two‐photon fluorescence microscopy; CLM, chemiluminescence microscopy; STORM, stochastic optical reconstruction microscopy. X‐ray spectroscopy methods (blue): μ‐XAFS, microbeam X‐ray absorption fine structure spectroscopy; XRM, X‐ray microscopy; XMT, and X‐ray microtomography. X‐ray diffraction methods (black): TEDDI, tomographic energy‐dispersive diffraction imaging; XRD‐CT, X‐ray diffraction‐computed tomography. Miscellaneous (orange): CL (or PL, photoluminescence), cathodoluminescence.^[^ ^133^, ^156^, ^172^, ^209^ ^]^

Although multiple resin printing is an exciting avenue, some restrictions might apply to GLACEs. First, the printed architectures' positioning accuracy should be controlled to stitch adjacent print fields together. The step will require maintaining similar RI between photoresins as RI changes can lead to aberrations compromising stitching. Other restrictions, particularly for GLACEs, are the inorganic loadings contained within the photoresin. Heavy inorganic payloads within the photoresist can lead to diffraction or scattering, compromising the pre‐GLACE architecture fidelity.^[^ ^24^ ^]^ Furthermore, the heavy inorganic loaded pre‐GLACE might not be transparent to light, causing light aberration (distortions of the focal position during printing), particularly when overlapping more than one material within the architecture (**Figure** **5**a). The strength of the aberrations depends on the RI contrast over the various architecture layers, where the light has to be crossed to reach the focal plane. Besides the inorganic photoresin payloads, the pre‐GLACE horizontal layers (slicing distance) and parallel lines (hatching distance) should be controlled to ensure microarchitecture fidelity. Curved microarchitectures can be challenging, because they require splitting the design into several layers with a minimal distance to avoid a staircase pattern. In this case, the minimal distance between the step sizes can lead to a reduced staircase. Additionally, decreasing the step size can minimize surface roughness (Tables 1 and 2), which is relevant for optical geometries (e.g., aberration lenses) requiring the assembly of dissimilar compositions. Additional parameters, like interfacial adhesion, are not considered in this discussion. However, they are known to play a role in the integrity of the 3D printed architectures, especially after annealing the pre‐GLACE architecture.^[^ ^173^ ^]^

## 8 Characterization of Single and Multi‐Composition GLACEs

Under the most favorable circumstances, where pre‐GLACEs are printed with spatially distributed materials, studying the crystal formation mechanism is important to generate insights into controlling GLACE architectures' crystallinity (sections 6 and 7). Interrogating GLACE architectures chemically and structurally may then require advanced characterization methods. Various methods can be used to analyze the chemical and crystal structure of GLACEs. These methods are essential for investigating the intricate details of mesoscale structures, which involve the interplay between aggregation and crystallization processes in GLACEs that can benefit optical functionality.^[^ ^24^ ^]^

An approach to understanding GLACE's chemical and structural characteristics to the finest level is X‐ray and neutron diffraction, which are potent techniques widely employed to probe solid materials. These methods offer detailed information on the atomic arrangement within crystals, assisting in determining crystal structures and identifying chemical compositions.^[^ ^206^ ^]^ Besides diffraction techniques, electron microscopy is crucial in characterizing inorganic solids. Scanning transmission electron microscopy (STEM) provides nanometer‐scale resolution, enabling researchers to visualize materials' morphology and structural features at the atomic level. This high‐resolution imaging technique is vital for studying nanometer inorganic solids' detailed crystal structure and morphology, offering valuable insights into their properties and behavior. However, in the case of GLACEs, STEM specimen preparation is required by using other techniques like focused ion beam milling.^[^ ^143^ ^]^ We can expect to interrogate larger structures shortly. Such steps will require approaches aided by computational methods capable of additionally processing spectroscopical and crystallographic information within hundreds of micrometers in space.

Understanding crystal formation kinetics will then require the use of in situ STEM. The collected information can be expected to be aided by quantum mechanical/molecular mechanical (QM/MM) approaches, which are instrumental in modeling and predicting the structures of inorganic solids.^[^ ^207^ ^]^ QM/MM computational tools can investigate solid materials' properties, defects, and transport mechanisms, providing valuable insights into their behavior at the atomic and molecular levels. However, limitations might be encountered as in situ STEM, and QM/MM requires a sizeable areal analysis to record a significant representation of the specimen area and powerful data processing and analysis capacities. Process analysis capacities can exponentially grow. For instance, incorporating electron energy loss spectroscopy (EELS) during in situ STEM analysis to corroborate chemical bond changes to understand molecular mechanisms upon the annealing of the pre‐GLACE.^[^ ^208^ ^]^

Insitu environmental scanning electron microscopy (ESEM) heating is an exciting choice for understanding cumulative changes of atoms/molecules within a few micrometers of resolution. Hence, it can be coined as a complementary method to in situ STEM. ESEM is expected to aid in understanding morphological changes during temperatures and atmospheric pressures on isothermal shrinkage of the pre‐GLACE,^[^ ^208^ ^]^ and it has excellent potential to observe crystallization by incorporating an electron backscatter diffraction analyzer (EBSD). However, precautions should be considered, as EBSD might not be fully comparable with ESEM pressure conditions, typically close to 500 Pa.

Other spectroscopies can be of absolute value for studying the mesoscale processes during the crystallization of GLACEs.^[^ ^209^ ^]^ X‐ray crystallography, as exemplified by the single crystal automated refinement (SCAR) method proposed by Viswanathan et al.,^[^ ^210^ ^]^ SCAR utilizes single crystal data to solve crystal structures of inorganic solids, employing data mining and machine‐learning techniques to automate the refinement process. This approach considers various structural features standard in inorganic materials, such as atom assignment and crystallographic site deficiency, facilitating the accurate determination of crystal structures. However, the challenge for GLACE is achieving single crystalline phase architectures. An overview of the different spectroscopic methods to understand the chemical and structural properties of GLACEs is summarized in Figure 5b. The methods presented in Figure 5b are adapted from Buurmans et al. ’s publication.^[^ ^209^ ^]^

The structural and chemical methods given in Figure 5b are techniques that can allow the understanding of crystal growth kinetics of GLACEs with various length scales. The methods are subdivided into four groups: vibrational spectroscopy methods (green‐colored: DORS, IRM, CRM, CARS, and SRS), electronic spectroscopy methods (red‐colored: CFM, CLM, 2PFM, and SNOM), X‐ray spectroscopy methods (blue‐colored: XRM, and XMT) and X‐ray diffraction methods (black‐colored: XRD‐CT). A miscellaneous group of techniques (orange‐colored) consists of CL (or PL).^[^ ^119^, ^211^ ^]^ Figure 5b provides the complete names of all abbreviations. The techniques include far‐field and near‐field optical methods. However, they have not been used or reported to interrogate the TPL‐printed GLACEs. We can encourage the community to use them to understand, e.g., the defect formation and its impact on the produced GLACE (Figure 4).

Overall, the advanced methods described could offer a deeper understanding of the GLACE microarchitectures' static or dynamic properties at the micrometer or nanometer scale. The methods mentioned are not exclusive. Other methods might be of absolute importance to understanding GLACE properties. The selection of the method might depend on the scientific question to be answered, which highly depends on the end application of the optical components. Optical components are described below.

## 9 Micro‐Optical Components

**Figure 6:**
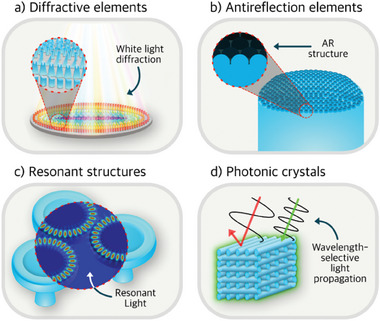
Schematic displaying light‐management strategies achieved with TPL‐printed GLACEs: a) diffractive elements, b) antireflection elements, c) resonant structures, and d) photonic crystals.

Light management is essential for controlling the performance of photovoltaics, optoelectronics, and optical systems. 3D‐pattern optical components into micro‐ and nanoarchitectures have provided geometric‐complex arrays, including pyramids, cones, spikes, domes, shells, bowls, and circular pores.^[^ ^212^ ^]^ As shown in Table 1, these geometries are often on the scale of UV and VIS to near IR wavelengths, presenting engineered electromagnetic properties in this spectral range that depend on their size, shape, and composition. Such structural designs allow light manipulation in reflection, extraction, and propagation. Surface‐patterned structures lack spatial precision, but extending the existing planar or quasi‐3D system enhances light propagation in the third dimension by introducing more degrees of freedom to the 3D architectures. A collection of light management strategies that can benefit from such 3D light control and propagation is provided in **Figure** **6**.

Light‐management strategies typically encompass scattering enhancements, antireflection techniques, resonance methods, and photonic crystal structures, as shown in Figure 6. This section will briefly discuss these strategies and the underlying principles of light‐matter interactions, emphasizing printed GLACEs. It should be noted that the strategies presented in Figure 6 do not distinguish between multi‐material integration within the same architectures and the use of different materials for distinct architectural features within a system, as this is an emerging field calling for discovery and further exploration.

### 9.1 Diffractive Elements

Using TPL it is possible to fabricate DOEs with unparalleled precision and detail,^[^ ^212^ ^]^ as shown in Figure 6a. DOEs can manipulate the phase of scattered waves, enabling the shaping of laser beams, such as lenses, deflectors, LED collimators, and fan‐out elements.^[^ ^214^ ^]^ Previous works have demonstrated the versatility of DOEs in complex structures, including subwavelength gratings, super‐oscillatory lenses, and biomimetic grating structures.^[^ ^213^, ^215^, ^216^, ^217^, ^218^, ^219^, ^220^ ^]^

In contrast to DOEs, scattering optical elements (SOEs) have proposed an alternative approach for light manipulation, leveraging multiple light scattering within a turbid layer.^[^ ^214^, ^221^ ^]^ SOEs offer light manipulation capabilities, including adjustments in intensity, polarization states, and spectral frequency, without the need for intricate optical design.^[^ ^221^, ^222^ ^]^ SOEs utilize disordered media and photopolymer‐based wavefront recorders, simplifying the fabrication process and paving the way for innovative light management solutions.^[^ ^223^, ^224^ ^]^

While DOEs excel in precision and detail, their fabrication often requires intricate 3D architectures and complex optimization processes.^[^ ^214^ ^]^ In contrast, SOEs offer a relatively straightforward step by utilizing inverse‐designed scatterers within a complex media to achieve desired optical functionalities.^[^ ^224^, ^225^ ^]^

When ordered arrays are desired, AM techniques, such as TPL, emerge as promising solutions to overcome the complexity of fabricating near‐perfect DOEs.^[^ ^215^ ^]^ Purtov et al. utilized TPL to fabricate grating structures composed of nanopillars, achieving diameters as small as 184 nm through precise laser power control during polymerization.^[^ ^216^ ^]^ Similarly, Cheng et al. demonstrated the creation of subwavelength gratings using TPL, focusing reflections in the near‐field with periods as short as 314 nm.^[^ ^213^, ^217^ ^]^ By enabling the direct fabrication of intricate 3D architectures, TPL simplifies the production process of DOEs,^[^ ^213^, ^215^, ^216^, ^217^, ^218^, ^219^, ^220^ ^]^ Although most works have focused on polymer materials, the flexibility of TPL facilitates the exploration of novel materials, including GLACEs and multi‐material configurations, expanding the research field and applications of DOEs.

Leveraging the capabilities of TPL, researchers can also explore novel multi‐material printing (Figure 5a), enabling the integration of GLACEs with other materials to tailor hybrid optical properties.^[^ ^216^ ^]^ For instance, Hsiung et al. demonstrated the fabrication of biomimetic grating structures using TPL, highlighting the potential of multi‐material configurations in improving optical functionalities.^[^ ^218^ ^]^ Strategies such as inorganic photoresins for multi‐printing GLACEs can offer innovative solutions in the field of telecommunications, biophotonics, and photovoltaics.^[^ ^213^, ^215^, ^216^, ^217^, ^218^, ^219^, ^220^ ^]^

### 9.2 Antireflection Elements

Minimizing reflection while maximizing transmission or absorption across a broad spectrum of incident light angles is crucial in photon‐related devices.^[^ ^226^, ^227^ ^]^ To achieve this goal, an array of patterned micro‐ and nanostructures have been effectively utilized as broadband and dispersive antireflective (AR) coatings or surfaces.^[^ ^228^ ^]^ A schematic antireflection coating is presented in Figure 6b.

In Figure 6b, the interaction of micro‐ and nanostructure arrays with light largely depends on the scale of the structures and periodicity of the array. For microarchitectured arrays, antireflection is achieved by trapping the incident light within the gaps of the array, especially when the depth and spacing of the individual units are comparable to the wavelength of the light. Significantly reducing reflectance, particularly within the visible spectra range. In the case of cone arrays where the dimensions of the periodic structures are smaller than the wavelength, the incident light interacts with the AR structure in a manner that gradually bends the propagation of light, resembling a surface with a gradient RI. This type of AR coating is based on structural architectures forming arrays with a gradient RI and offers broadband, omnidirectional, and polarization‐insensitive antireflective capabilities.^[^ ^229^, ^230^ ^]^

Integrating GLACEs can help to advance AR in optical elements. While research has primarily focused on polymer‐based AR structures, recent studies have demonstrated the feasibility of AM in fabricating intricate AR patterns with GLACEs. For instance, a study utilized TPL to fabricate moth‐eye AR structures on lens surfaces, achieving remarkable antireflective properties.^[^ ^231^ ^]^ The moth‐eye structure, featuring dimensions of 470 nm width, 360 nm height, and an 800 nm pitch stripe, exhibited a low reflectance of 0.005% and a significant improvement in coupling loss at a wavelength of 1550 nm. Despite these promising results, further research is needed to optimize AM parameters and material compositions for GLACEs, ensuring scalability and cost‐effectiveness in AR coating fabrication.^[^ ^231^ ^]^

### 9.3 Resonant Architectures

#### 9.3.1 Mie Resonators

Mie's theory primarily addresses light scattering in dielectric particles with sizes comparable to the optical wavelength, revealing that high RI nanoparticles can exhibit scattering increases at resonances.^[^ ^232^ ^]^ Although the original theory applies to spheres and ellipsoids, Mie resonances are also present in particles of varied shapes. The nanostructure parameters determine the resonant wavelength and bandwidth. Mie resonators are key in designing metamaterials, which require arrangements with comparable scales to vacuum light wavelengths to attain collective properties governed by the optical properties of individual units.^[^ ^233^ ^]^ An application can be found in large‐scale all‐dielectric metamaterial reflectors comprising Si cylinder Mie resonators with reflectance exceeding traditional metallic mirrors.^[^ ^234^, ^235^ ^]^ Additionally, Mie resonators with low‐loss electromagnetic responses for efficient broadband wavefront manipulation provide a design approach for a new class of compact optical devices. Moreover, Mie modes can enhance the Purcell factor, offering further advantages in applications requiring high emission rates, particularly when combined with their low‐loss characteristics, making them ideal for efficient light‐matter interactions in photonic devices.^[^ ^236^ ^]^ Exploring low‐loss dielectric architectures and metasurfaces that support strong resonances marks a significant advancement in nanophotonics, with applications in areas like biosensing, quantum computing, and integrated photonics.^[^ ^237^ ^]^

GLACEs, which combine glass and ceramics, show great potential for advancing micro‐ and nanophotonics. Traditional fabrication methods face challenges in achieving precise resonator architectures, but GLACE printing offers a promising solution. The RI of GLACEs can vary widely, typically ranging from ≈1.5 for glass to as high as 2.5–2.8 for ceramics like ZrO₂, SrZrO₃, or TiO₂. Several factors, including resin formulation, grain formation, and 3D printing parameters, such as laser power and scan speed, influence the effective RI. These conditions can significantly affect the effective RI. E.g., with its amorphous nature, glass can minimize crystal boundary effects, while ceramics generally offer higher refractive indices. Recent advancements in 3D‐printable microwave and Fano resonances further demonstrate the potential of AM in fabricating complex Mie resonator geometries.^[^ ^238^ ^]^ Nevertheless, further research is required to optimize printing parameters and develop high‐index GLACE materials for photonics applications.

#### 9.3.2 Guided Mode Resonance

Guided Mode Resonance (GMR) occurs when incident light couples into a waveguide mode, leading to strong reflection and transmission effects. Resonant modes can be generated by periodic structures, such as diffraction gratings above light‐absorbing layers.^[^ ^238^, ^240^ ^]^ However, GMR can also be excited through alternative methods, such as prism coupling, where light is directed into the waveguide using a prism.^[^ ^241^, ^242^ ^]^ The fundamental principle involves matching the incoming light's wavelength with the guided mode's wavelength, facilitating efficient light‐matter interaction. By tailoring the waveguide and surrounding structures, resonant behavior can be precisely tuned for specific wavelengths. Advances in 3D printing, particularly in ceramics, allow for the fabrication of complex micro‐ and nanoscale geometries required to engineer these resonances. In the following sections, we discuss Fabry‐Perot resonances and Whispering Gallery Mode (WGM) resonators, which can also benefit from such 3D fabrication techniques.

##### Fabry‐Pérot Resonances

Fabry–Pérot (F–P) resonances occur in thin films where two mirrors are separated by a dielectric material, which serves as the cavity medium. Within this cavity, constructive interference at certain wavelengths produces either a transmission or reflection peak. The reflectivity of these F–P cavities (RFP) can be described by the following equation:^[^ ^239^ ^]^

(1) $$R_{FP}=\frac{2R\cdot \left(1-\cos\delta \right)}{1+R^{2}-2Rcos\delta }$$

where R is the reflectivity of the film, δ is the phase difference between each successive reflection. Selecting a cavity length that resonates at wavelengths corresponding to red, green, or blue (RGB) colors results in the reflection of their complementary colors, cyan, magenta, or yellow (CMY).^[^ ^240^, ^243^, ^244^, ^245^ ^]^

GLACEs can aid in producing F‐Ps with highly complex designs. For example, Lin et al. investigated the optical properties of silicon carbide (SiC) F‐P cavities, demonstrating their potential for highly selective light absorption and spectral tunability in the visible light range.^[^ ^240^ ^]^ The study highlights the optimal performance of SiC on Al substrates for harsh environments. It discusses the thickness‐dependent spectral shift of the resonators, with theoretical analysis closely matching simulation results and promising advancements in color filters and photovoltaic devices.

##### WGM Resonators

WGM resonators, supported by dielectric microarchitectures like spheres, toroids, and rings, confine light through total internal reflection at their periphery (see Figure 6c). The allowed modes can be described by the expression:^[^ ^246^ ^]^

(2) $$\lambda_{res}=\frac{2\pi r\times n_{eff}}{m},m=1,2,3$$

where λ_*res*_ is the resonant wavelength, r is the ring radius, *n_eff_* is the effective RI, and *m* is an integer related to the mode order. At resonance, the electromagnetic field forms a standing wave pattern within the dielectric architectures. The quality (*Q*) factor of a WGM resonance is dependent on the material and geometry.^[^ ^241^, ^246^ ^]^

WGM resonators can be fabricated from various materials, including silicon and organic halide perovskites.^[^ ^242^, ^247^ ^]^ An attractive avenue for WGMs is GLACEs, which offer opportunities for fabricating WGM resonators with tailored geometries, particularly leveraging their resistance to temperature and corrosion compared to traditional polymer‐based materials. Wen X. et al.^[^ ^135^ ^]^ designed a strategy for 3D GLACE architectures using TPL. The authors demonstrated the production of nanoscale glass WGM at different resonant wavelengths. The produced WGM architectures exhibited the possibility to trap and increase light absorption, making them ideal for integrating into other devices, such as single chips, enabling multiplex optical operations.^[^ ^248^ ^]^

### 9.4 Photonic Crystals

Photonic crystals (PhCs) are shown in Figure 6d. This periodic variation creates a photonic bandgap, a range of wavelengths at which light propagation is forbidden due to destructive interference. This bandgap enables precise control over light, with applications in light bending,^[^ ^249^ ^]^ optical diodes,^[^ ^250^ ^]^ negative refraction,^[^ ^251^ ^]^ and self‐collimating systems.^[^ ^252^ ^]^ The behavior of light within PhCs can be more complex than simple gratings, as they influence light in both reflection and transmission, depending on the wavelength relative to the periodicity of the structure. Generally, the bandgap occurs at wavelengths on the order of or slightly longer than the periodicity of the crystal, but for metamaterials, the wavelengths are typically much smaller than the periodicity. The presence and position of this bandgap are deducible through the Bragg–Snell equation:^[^ ^253^ ^]^

(3) $$m\lambda =2d\sqrt{n_{eff}^{2}-sin^{2}\theta }$$

where *λ* represents the wavelength of the light of the photonic bandgap location, *m* is the Bragg reflection order, *d* is the PhC's lattice constant, *n_eff_* its effective RI and θ the angle of light incidence relative to the surface is normal. The *n_eff_* of a PhC can be a function of the wavelength and varies depending on the geometry. Therefore, a mathematical expression for *n_eff_* needs to be evaluated for each case. Notably, for a 1D simple PhC made of two components (e.g., colloids and air), *n_eff_* is calculable by the formula:^[^ ^253^ ^]^

(4) $$n_{eff}=\sqrt{n_{1}^{2}f_{1}+n_{2}^{2}f_{2}}$$

where *f*_1_ and *f*_2_ are volume fractions and *n*_1_ and *n*_2_ are refractive indices of the constituent materials.^[^ ^253^ ^]^ Research into PhCs spans various applications, from sensing to optical communications.^[^ ^254^, ^255^ ^]^ Additionally, PhCs have been utilized in beam power splitters^[^ ^256^ ^]^ and polarization beam splitters^[^ ^257^, ^258^ ^]^ essential for integrated photonics and communication systems, where design optimizations have achieved high polarization extinction ratios.^[^ ^259^, ^260^, ^261^ ^]^

The development of 3D printing has opened new avenues for fabricating PhCs with high precision and complexity. TPL GLACEs present the potential for PhC fabrication. Vyatskikh et al.^[^ ^118^ ^]^ showcased the production of a high RI 3D TiO_2_ PhC featuring a partial photonic bandgap. Defects in the PhC have impacted the achievement of complete photonic bandgaps. A way forward to circumvent such defects is tailoring the PhC surface to avoid unwelcome optical features. The continuous evolution of 3D printing and TPL technologies promises to increase the capabilities and applications of PhCs. Advances in manufacturing are crucial for realizing the next generation of optical devices. Therefore, in the next section, we assess the various optical devices and reflect upon the integration of GLACEs.

## 10 Advanced Micro‐Optical Devices

**Figure 7:**
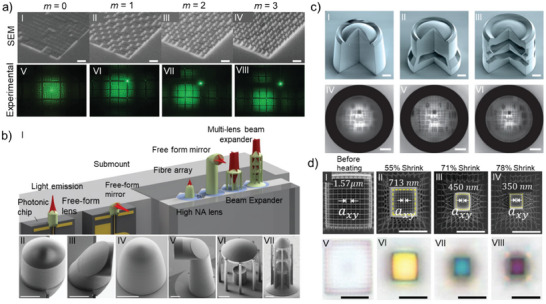
a) Deflection of the energy distribution for different m orders. (I‐IV) SEM images (scale bar: 5 µm), and (V‐VIII) experimental holographic images. Reproduced with permission from.^[^ ^264^ ^]^ b) (I) Schematic elements. (II‐V) Free‐form lens and (VI, VII) Beam expander. All scale bars, 20 µm. Reproduced with permission from.^[^ ^29^ ^]^ c) (I‐III), SEM images of the singlet, doublet, and triplet cut/printed lenses. (IV‐VI) Test chart imaged by the printed lenses. Scale bars, 20 µm. Reproduced with permission from.^[^ ^28^ ^]^ d) SEM images (I‐IV) and corresponding brightfield reflection‐mode optical micrographs of the PhC (V‐VIII). Reproduced with permission from.^[^ ^67^ ^]^

Micro‐optical devices have been designed for unique applications based on a deeper understanding of optical principles. As discussed in the previous section, functional optics can be achieved through the components’ material, structural, and 3D‐printed geometrical design. Hence, researching manufacturing methods for functional optics has become a trending topic in diverse fields. Nevertheless, the structural complexity and multi‐material distribution of advanced micro‐optical devices demand advanced manufacturing technology, such as AM, which has demonstrated its potential to produce multi‐scale, multi‐material, and multi‐functional optical systems for imaging, sensing, displaying, and light‐modulating.^[^ ^126^, ^262^, ^263^ ^]^ Despite the impressive examples of polymers in the literature on micro‐optical devices, GLACEs might revolutionize this field by offering chemical and mechanically resilient optical components. Therefore, this section aims to illustrate the potential fields in which TPL, is forging new frontiers in advanced micro‐optics, foreshadowing a future where the full spectrum of optical devices benefits from GLACEs (**Figure** **7**).

### 10.1 Diffractive Optics

Diffractive Optics focuses on utilizing the properties of DOEs to transform light efficiently. DOEs, composed of various zones, use diffraction to interfere with light, producing images through coherent superposition. Traditional 2D lithography techniques, which limit the production of freeform topographies, are transcended by TPL's 3D flexibility and submicron precision.^[^ ^215^ ^]^ TPL facilitates the production of intricate microarchitectures in space, including gray‐level encoded microarchitectures^[^ ^265^ ^]^ and multi‐level architectures.^[^ ^266^, ^267^ ^]^ TPL's capabilities also extend to producing binary radial DOEs^[^ ^268^ ^]^ and four‐level micro‐relief DOEs,^[^ ^269^ ^]^ demonstrating its versatility. The printing technology also enables the production of inverse‐designed thin circular grating‐like structures atop fibers for wavefront modifications^[^ ^270^ ^]^ and stacked DOEs to correct field‐dependent aberrations in planar lenses.^[^ ^271^ ^]^ Additionally, TPL facilitates the fabrication of continuous surface Fresnel micro‐lens arrays for 3D focal field engineering.^[^ ^272^ ^]^

Overall, the integration of TPL techniques in the fabrication of DOEs has heralded new advancements in diffractive optics, offering precise manipulation of light across various wavelengths to generate desired patterns or structural beams. Recent endeavors in this domain have created grating and diffractive structures. Innovations include the production of nanopillar‐based grating structures with diameters as fine as 184 nm for near and far‐field focusing applications and the development of super‐oscillatory lenses with asymmetrical gratings.^[^ ^213^, ^216^, ^217^, ^273^ ^]^ TPL also facilitated the design of biomimetic structures and phase masks for wavelength separation and sunlight splitting for photovoltaics.^[^ ^218^, ^219^ ^]^

The versatility of TPL in diffractive optics is illustrated by highlighting H. Wang et al.’s achievements with the printing of a DOE with sub‐pixel level modification.^[^ ^264^ ^]^ Figure 7a illustrates the characterization of such holographic DOEs designed to manipulate holographic images by varying grating orders (m = 0, 1, 2, and 3). Figure 7a (I‐IV) displays various scanning electron microscope (SEM) images at a 50° oblique angle, showcasing the DOEs' surface structure. The *m* = 0 has a flat top. In contrast, those with higher *m* values feature blazed facets with progressively steeper slopes, as intended, ensuring the phase information is accurately preserved through consistent height differences between adjacent phase blocks. Figure 7a (V‐VIII) presents holographic images for each grating order. From the previous example, it is fair to say that hologram applications can benefit from integrating high‐refractive‐index GLACEs. Furthermore, H. Wang et al.’s^[^ ^264^ ^]^ research can benefit from multi‐RI DOEs using multi‐material printing (Figure 5a), which can display a more comprehensive color range with higher detail. These advancements emphasize the role of AM of GLACEs in exploring the expansive potential of diffractive optics across the X‐ray, visible, and infrared ranges. The precise fabrication of arbitrary surface topographies, combined with structural stability and material compatibility, positions AM as an indispensable tool for applications in augmented reality, virtual reality, diffractive neural networks, and optical computing, marking a significant leap forward in the development of advanced micro‐optical devices.

### 10.2 Fiber and Waveguide Applications

Fiber and waveguide optics have benefited immensely from TPL's ability to fabricate intricate free‐form waveguide configurations. TPL's versatility allows for creating 2D/3D waveguide trajectories embedded in a low‐index cladding material for optical interconnects.^[^ ^274^ ^]^ Innovations include “light cage” hollow‐core waveguides for sensing^[^ ^275^ ^]^ and solid‐core waveguides using different photoresists or controlled polymerization for core and cladding areas.^[^ ^10^, ^276^, ^277^ ^]^ TPL has propelled advancements in optical communications, with applications ranging from single‐mode waveguides for interconnects^[^ ^278^ ^]^ to hybrid devices combining 2D and 3D elements for light coupling.^[^ ^279^ ^]^ For example, Schröder et al.^[^ ^280^ ^]^ spearheaded this journey by directly demonstrating the fabrication of single‐mode polymer waveguides on integrated circuits, illustrating the compatibility of TPL with high‐density printing and CMOS technology. Following this seminal work, Nguyen et al.^[^ ^281^ ^]^ expanded the scope by employing TPL to create single‐mode embedded waveguides from a unique material, achieving remarkable RI contrast and facilitating the production of multilayer waveguides. Furthermore, Schumann et al.^[^ ^279^ ^]^ produced waveguide bridges that act as polarizing elements within an integrated circuit. This innovation ensured mechanical stability and minimized transmission loss, showcasing TPL's capacity to substitute traditional fabrication techniques.

In another research, Landowski et al.^[^ ^282^ ^]^ further advanced TPL applications by optimizing out‐of‐plane coupling in high‐density optical chips, leading to coupling efficiency alongside reduced insertion and transmission loss. Later, P.‐I. Dietrich et al. explore and demonstrate a toolbox of essential beam‐shaping elements that can be universal building blocks for hybrid multi‐chip systems.^[^ ^29^ ^]^ These elements are illustrated in Figure 7b (I), and SEM images of 3D‐printed elements are shown in Figure 7b (II‐VII). Based on a single refractive surface, P.‐I. Dietrich et al. work^[^ ^29^ ^]^ achieved free‐form lenses for beam‐shaping elements (Figure 7b (II, IV)) and free‐form mirror surfaces for reflecting elements (Figure 7b (III, V)). In addition, intricate architectures are possible, comprising combinations of convex and concave lenses for beam expansion (Figure 7b (VI)) or high‐performance multi‐lens assemblies (Figure 7b (VII)). The beam‐shaping elements can be designed for operation in air or in a low‐index cladding material, which reduces reflection and protects the optical surfaces from environmental influences.^[^ ^283^ ^]^

Additionally, the field of holography witnessed a significant advancement through Zheng et al.^[^ ^284^ ^]^ who employed TPL to generate a static optical phased array. This development achieved broad diffraction angles, enabling pixel‐wise manipulation for holographic applications. Optical components for phase control and spectral selection also benefitted from TPL. Belle et al.^[^ ^285^ ^]^ demonstrated the fabrication of hollow waveguide arrays acting as quarter‐wave plates capable of large‐scale production via field stitching. Similarly, Goraus et al.^[^ ^286^ ^]^ and Wei et al.^[^ ^287^ ^]^ utilized TPL to create Bragg grating waveguides and phase‐shifted Bragg gratings, respectively. These components enhanced direct fiber coupling and sensitive ultrasound detection, highlighting TPL's role in producing highly specialized optical devices. Last, Li et al.^[^ ^288^ ^]^ contributed to this innovative landscape by fabricating waveguide 1D PhCs using TPL, offering tunable reflectance and potential as specular reflectors in the near‐infrared spectrum.

Collectively, these contributions illustrate the transformative impact of TPL in fiber and waveguide optics, paving a new era of photonic devices characterized by unprecedented precision, flexibility, and material diversity. Shortly, it is expected that GLACEs can help diversify the optical element's RI. This can also be the case with optical components, e.g., cladding material can be achieved with multi‐material 3D printing, reducing the refractiveness of an individual optical interface (Figure 7b (VII)).

### 10.3 Imaging Applications

Imaging optics has evolved significantly with the advent of TPL, enabling the miniaturization of imaging systems. TPL's precision in fabricating micro‐sized optics is key to high‐performance and complex micro‐optical systems, including microlens arrays for imaging.^[^ ^289^ ^]^ This technology also aids aberration correction, as demonstrated by T.Gissibl et al.^[^ ^28^ ^]^ who optimized singlet, doublet, and triplet lenses to correct monochromatic aberrations (Figure 7c). The lenses were produced on a glass substrate to achieve a broad field of view (FOV) of 80°. Figure 7c (I‐III) showcases SEM images of the manufactured singlet, doublet, and triplet lenses. Figure 7c (IV‐VI) directly assesses the lenses' optical performance by presenting images of the USAF 1951 resolution test chart captured through each lens type. A key parameter in lens design is the numerical aperture (NA), which defines the ability of a lens to gather light and resolve fine details at a given focus. Higher NA values allow the lens to collect more light from a wider angle, improving resolution and brightness. For example, applications in endoscopy have seen remarkable advancements, such as high‐NA aspheric microlenses for deep brain imaging^[^ ^290^ ^]^ and coherent beam combiners for efficient two‐photon imaging.^[^ ^291^ ^]^

Novel side‐facing free‐form micro‐optics on single‐mode fibers have led to advancements in optical coherence tomography.^[^ ^292^ ^]^ The lens‐in‐lens design^[^ ^293^ ^]^ and phase masks for 3D fluorescence microscopy^[^ ^294^ ^]^ exemplify the diverse imaging optics applications. Kim et al.^[^ ^295^ ^]^ and Thiele et al.^[^ ^69^ ^]^ have pushed the boundaries of imaging resolution and FOV by developing high‐resolution imaging systems and CMOS image sensors inspired by the eagle. Their work showcases TPL's customization capabilities in creating sophisticated imaging systems. The biomimetic designs by Dai et al.^[^ ^296^ ^]^ and Hu et al.^[^ ^291^ ^]^ including microfluidic‐assisted 3D printed compound eyes and logarithmic profile ommatidia, demonstrate TPL's adaptability. These innovations provide panoramic views and innovative solutions to defocusing issues.

In other work, which can aid in highlighting GLACE multi‐material capability, Ren et al.^[^ ^297^ ^]^ and Balli et al.^[^ ^298^ ^]^ have significantly advanced lens technology by fabricating hybrid lenses for achromatic focusing. The achromatic lenses have been designed to minimize chromatic aberration, which occurs when different wavelengths of light are focused at varying points, leading to color fringing and blurring. These lenses achieve sharper imaging across a broad spectrum of light by combining materials with distinct dispersion properties

GLACE strategies can tailor high RI materials with low dispersion properties without compromising optical quality or precision.^[^ ^297^, ^298^ ^]^ Additionally, in overcoming throughput challenges, Ristok et al.^[^ ^123^ ^]^ have produced large‐scale lenses with uniform RI, marking a significant advancement in TPL's application for creating larger optical components, which can be a promising direction for GLACEs.

### 10.4 Color Applications

Color optics involves manipulating and controlling light's color properties, typically using materials and structures that interact with specific wavelengths. In the context of TPL, this can involve using photopolymers, ceramics, or nanoparticles to create devices that filter, reflect, or transmit light in a wavelength‐selective manner. Among the diverse architectures, pillars are at the forefront in color optics, and they are lauded for their simplicity. By adjusting the height of these pillars, researchers can span a wide range of visible colors, showcasing their functionality as color filters.^[^ ^220^ ^]^ Pillars can consist of a multi‐material composition in a multilayer approach, where their inclusion by TPL can be fundamental. The versatility of nanopillars extends to the creation of full‐color and grayscale artworks, holographic color prints, and even optical moiré effects.^[^ ^299^, ^300^, ^301^ ^]^

Going beyond pillar architectures, TPL has been applied to diffraction gratings to generate angle‐dependent colors. This method allows for control over the grating's geometry, revealing hidden color data and probing the color attributes of bi‐gratings.^[^ ^302^, ^303^ ^]^ Notably, TPL facilitates post‐production color adjustments, as demonstrated in studies involving stretching ratings,^[^ ^304^ ^]^ which can pave the way for dynamic color management.

Regarding coloration, the woodpile PhCs architecture is another TPL milestone, capable of rendering colorful, arbitrarily shaped 3D objects. Despite challenges in attaining complete bandgaps within the visible spectrum due to TPL's resolution constraints,^[^ ^305^ ^]^ solutions like heat shrinking processes have been shown by Liu et al. to yield slow light modes,^[^ ^67^ ^]^ fthe color fidelity of the fabricated architectures. Figure 7d I‐IV shows SEM and corresponding optical micrographs of the structures after 12, 17, and 21 min of heating at 450 °C, clearly showing a lateral shrinkage of 55% (*axy  =  713 nm*), 71% (*axy  =  450 nm*), and 78% (*axy  =  350 nm*), respectively. The color is not observed before heating (Figure 7d V). Colors appear, shifting from yellow to blue and purple for linear shrinkage values of 55% (≈600 nm), 71% (≈508 nm), and 78% (≈445 nm) (Figure 7d VI‐VIII).

To replicate intricate color schemes, multi‐material TPL has been used to create microscale droplets and multilayer biomimetic structures.^[^ ^306^ ^]^ Such an application mimics the iridescent hues in butterfly wings and highlights TPL's potential for designing complex color effects with high fidelity. Additionally, TPL's impact extends to developing miniature spectrometers,^[^ ^307^ ^]^ which operate within the visible spectrum. This example underscores the practicality of TPL in crafting essential optical instruments. Despite these successes, TPL's primary challenge lies in its relatively low throughput, attributed to the serial nature of its patterning process. Furthermore, in GLACEs, post‐processing thermal treatment and specific integration strategies are expected. Future directions may include implementing parallel TPL systems for mass production.^[^ ^308^ ^]^ However, post‐processing steps to achieve optical‐grade GLACEs should be developed.

### 10.5 Optical Storage Based on Quantum Optics

Quantum optics and optical storage delve into the role of on‐chip photonic circuits at the quantum level, which is crucial for future quantum information processing. Photonic elements like PhCs, resonators, and waveguides are key, and assembly strategies in quantum photonic devices should be explored. Schell et al.^[^ ^311^ ^]^ demonstrated a combined structure with interconnects, including resonators and emitters, incorporating nanodiamonds with nitrogen‐vacancy centers. This structure showcased active quantum functionality for single‐photon generation and routing via waveguides.

Within quantum optics, single‐photon sources have seen advancements like Fischbach's^[^ ^312^ ^]^ quantum light source, which is integral for transferring quantum light to external optics. Additionally, quantum emitters embedded in TPL‐fabricated polymer waveguides^[^ ^313^, ^314^ ^]^ simplify excitation and photon collection. Furthermore, electromagnetically‐induced transparency (EIT) in light cage structures^[^ ^315^ ^]^ opens possibilities for quantum storage and nonlinear optics applications. The innovation in active optical elements by F. Davidson‐Marquis et al.^[^ ^315^, ^316^ ^]^ using TPL‐manufactured metasurfaces introduces a toolkit for dynamic light control, essential for quantum experiments requiring precise optical manipulations.

**Figure 8:**
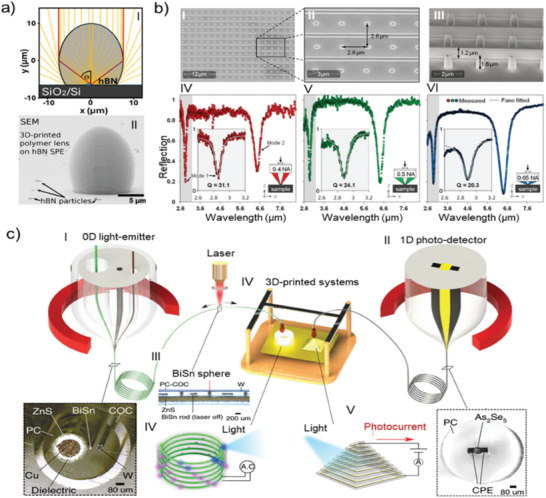
(a) (I) Schematic of the lens coupled to hBN nanoparticle. (II) SEM image of a lens printed on an hBN nanoparticle. Reproduced with permission from.^[^ ^73^ ^]^ b) (I‐III) SEM images of a hybrid metasurface. (IV‐VI) Experimental reflection spectra for different objectives. Reproduced with permission from.^[^ ^309^ ^]^ c) Multi‐material filament inks for 3D printed functional systems. (I) 0D light‐emitting and (II) 1D light‐detecting filaments. (III) BiSn spheres to form programmable‐placed pixels within the light‐emitting filament. (IV) Electrically activated 3D systems capable of spatial (V) light‐emission and light detection. Reproduced with permission from.^[^ ^310^ ^]^

In another work, J. A. Preuß et al. manufactured elliptical microlenses to enhance the free‐space collection efficiency of light emitted from single‐photon emitters embedded in hexagonal boron nitride (hBN) nanocrystals.^[^ ^73^ ^]^ By designing the lenses to have specific elliptical shapes, they successfully collimate the emitted light, minimizing divergence and maximizing photon collection efficiency. These lenses are designed to operate across the visible spectrum, offering a novel approach to optimizing light collection. **Figure** **8**a I shows the polymer lens's design sketch, detailing the expected ray tracing. The emission from a point source at the bottom interface is projected as a collimated beam. An hBN nanoparticle, depicted in blue (not to scale), demonstrates the sample geometry, indicating a highly efficient, low‐divergence light emission facilitated by the lens design. Figure 8a II: Presents an SEM image of an individual polymer lens printed onto an hBN particle. Contributions from researchers^[^ ^126^, ^291^, ^317^ ^]^ have spotlighted TPL's adeptness in miniaturizing optical elements, which are now seamlessly integrated into optical fibers and imaging systems, enhancing the functional capabilities of quantum optical instruments.

The work led by M. Blaicher et al.^[^ ^318^ ^]^ has ingeniously applied TPL in photonic wire bonding, solving intricate challenges related to integrating micro‐scale lasers and optical circuits, thereby facilitating complex yet efficient photonic networks for quantum computation. Exploratory work integrating complex optical properties, as demonstrated by refs. [319, 320] enriches the toolbox for encoding and processing quantum information, exploiting TPL's finesse in crafting devices that leverage the intricate properties of light for advanced quantum applications.

Previous work in 3D optical data storage by refs. [321, 322, 323] has dramatically expanded data capacity, showcasing TPL's transformative potential in transcending conventional storage limits, thereby heralding a new age of data density and retrieval efficiency. The efforts by Yanez et al.^[^ ^324^ ^]^ in achieving unparalleled storage densities illuminate TPL's capability to harness novel materials for next‐level data compaction, paving the way for ultra‐dense, efficient storage solutions crucial for future computing technologies. We expect that in the new future GLACEs, custom‐photoresists will achieve similar or, if not even better, characteristics than the TPL polymer counterparts.

### 10.6 Topological Optics

Topological optics is a field that explores the interaction of light with structured materials. This area is closely related to the study of topological insulators and involves the manipulation of light in ways that are robust against defects and disorder. The TPL opens possibilities for creating optical devices whose responses are guided by topography. This includes waveguiding and light‐trapping elements. TPL can contribute to this field by facilitating the creation of complex geometries, which are essential for exploring phenomena such as bound states in the continuum (BIC). Previous research^[^ ^325^, ^326^ ^]^ laid the groundwork for BIC in photonic systems, demonstrating TPL's ability to construct localized states that do not couple to far‐field radiation, thus expanding the frontiers of light‐matter interaction. Significant strides in plasmonic BIC modes have been shown through the efforts of Liang Y. et al.^[^ ^327^, ^328^ ^]^ who harnessed TPL to produce architectures that act as perfect absorbers. These architectures showcased remarkable field enhancement and high *Q* factors.

Aigner A. et al.^[^ ^329^ ^]^ illustrated TPL's role in breaking the out‐of‐plane symmetry in plasmonic BIC metasurfaces. One example of a BIC metasurface is shown in Figure 8b.^[^ ^309^ ^]^ Figure 8b (I‐III) depicts SEM images of the hybrid metasurface from both a top view and a 45° angle perspective. Figure 8b (IV‐VI) shows the experimental reflection spectra obtained using different NA reflective objectives (NA = 0.4, 0.5, 0.65). Color dots mark the reflection spectra, and the insets schematically represent the various NA objectives used in the measurements.

In retrospection and reflecting upon multi‐material GLACEs (Figure 5a), BIC metasurface properties can be modified through multi‐material composition to achieve perfect absorption. In addition, GLACEs can be selected depending on the required gain and loss of the optical medium. Incorporating gain and loss materials and quantum emitters within topologically designed architectures^[^ ^313^ ^]^ can open new avenues for exploring non‐Hermitian physics and realizing quantum states.

### 10.7 Optics and Electronics

Integrating optics with electronics, or optoelectronics, represents a new frontier in technological advancements, where devices convert light into electrical signals and vice versa (Figure 4b). This field encompasses a wide range of applications, from infrastructure and military to medical industries, including key devices like waveguides, LEDs, laser diodes, photodetectors, and solar cells.^[^ ^128^, ^330^, ^331^, ^332^ ^]^ For example, Li et al.^[^ ^333^ ^]^ demonstrated TPL's utility in fabricating hollow waveguides from polylactic acid (PLA), achieving minimal loss at terahertz frequencies. Further advancing the complexity and precision of waveguide fabrication, another research^[^ ^334^ ^]^ utilized PolyJet 3D printing technology to construct waveguides based on the Kagome PhC architecture. The work exemplifies TPL's capacity for meticulous control over sophisticated waveguide designs and functionalities.

Due to the potential of terahertz waveguide technology, Li et al.^[^ ^335^ ^]^ employed SL to develop hollow‐core Bragg waveguides. Li et al.^[^ ^335^ ^]^ efforts highlight the adaptability of TPL in producing multi‐material devices with exact multilayer configurations for enhanced optical inspection capabilities. For example, to reduce optical losses, TPL could offer multi‐material printing tailored to free‐form cladding and waveguide fabrication. In another study, Udofia et al.^[^ ^336^ ^]^ introduced an innovative approach to optical customization using a soft, stretchable thermoplastic polymer, demonstrating TPL's adaptability for printing waveguides on 3D conformal surfaces. This technique facilitates the integration of optoelectronics in novel configurations.

For light emission, Kong et al.^[^ ^337^ ^]^ reported the fabrication of quantum dot LEDs through extrusion‐based 3D printing, underlining TPL's significant role in merging active nanoelectronics with diverse materials to forge new LED designs. Then, Loke et al. printed multi‐material interfaces (see Figure 8c) with structured filaments for light emission and detection.^[^ ^310^ ^]^ Figure 8c I illustrates a 0D light‐emitting filament. Microstructured filaments contained a metallic BiSn core, electrically conducting W, electroluminescent ZnS, and an insulating polycarbonate (PC) cladding. This architecture is aimed at tailoring electroluminescence functionality to a 3D‐printed object. Figure 8c II depicts the creation of a 1D light‐detecting filament, highlighting a multi‐material composition that includes semiconducting arsenic‐selenide (As_2_Se_5_), conducting polyethylene (CPE), and insulating PC. Using TPL, 3D multi‐material compositions might be integrated into complex systems. Figure 8c III shows the spatially‐resolved laser‐induced formation of BiSn spheres within the light‐emitting filament. Figure 8c IV illustrates the setup that allows for the tailored formation of electrically activated 3D systems capable of emitting and detecting light. Figure 8c V showcases the final printed objects demonstrating the light‐emission and light‐detection capabilities from the entire architectures of the 3D‐printed systems. Exploring other materials and multi‐material compositions for 3D printing will be important in the coming decades, especially for micro‐LEDs, for which recently thermoplastics have been shown as encapsulation materials for perovskite nanocrystals, leveraging TPL's potential in crafting light‐emitting devices for lighting and display technologies.^[^ ^338^ ^]^

In short, 3D printing technology, particularly TPL, continues to evolve with the exploration of GLACEs for optoelectronics. The ability to fabricate high‐precision architectures could revolutionize the field of optoelectronics, leading to devices with improved efficiencies and novel functionalities. The ongoing development of TPL‐compatible GLACEs will be crucial for realizing the full potential of 3D‐printed optoelectronics, marking a significant step forward in the fusion of AM with electronic and photonic systems.

## 11 GLACEs: a Road Map

**Figure 9:**
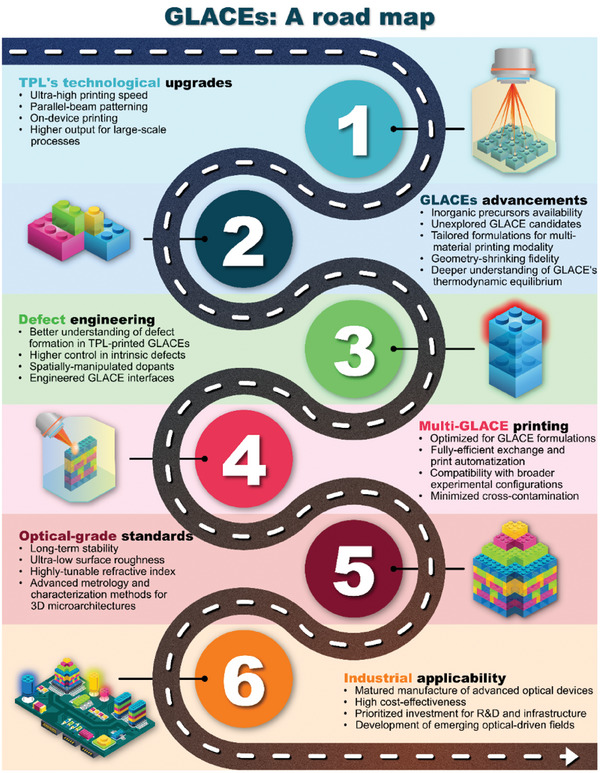
Technological roadmap for TPL 3D printing of GLACEs, depicting the expected challenges and future outlook of Next‐Gen additively manufactured micro‐optics, and envisioned development of TPL‐printed multi‐GLACEs to bridge micro‐optical manufacturing.

**Figure** **9** shows a schematic technological roadmap focusing on the expected challenges and future outlook GLACEs may encounter given the previously reviewed state‐of‐the‐art. The roadmap emphasizes the need for TPL technological upgrades alongside much‐required advances for photoresist development with the necessary tunability to minimize defects in single or multi‐material GLACE elements, fulfilling optical quality standards of the current transitioning industry.

## 12 Conclusions

Commencing with the technological status of TPL, assessed in Section 2, we disclosed its enormous potential due to the ultra‐high resolution achieved in different target materials. Yet, as mentioned in Table 1, we remark on the ongoing necessity of overcoming TPL's inherent slow manufacturing processing compared to other AM methods (e.g., PµSL, µ‐SLS, and DIW). Although direct laser writing approaches such as TPL are known for individual patterning of architectures, resulting in low polymerization scan speeds and small printing areas, exploiting parallel‐beam pattering may enable higher throughput and higher printing speeds. For instance, Shunhua Yang and co‐authors^[^ ^339^ ^]^ explored using a DMD and a microlens array (MLA) to generate thousands of individually controlled laser beams (i.e., individual on‐off switching and intensity‐tuning capability), achieving high uniformity without compromising the ultra‐high resolution (i.e., below 200 nm).

As a successive challenge, assessed in Section 3, micro‐optical materials must comply with diverse paramount characteristics such as high RI tunability (Table 2). In this direction, GLACEs have demonstrated to satisfy some characteristics (Table 3) and even set higher standards for long‐term resilience (e.g., high resistance to harsh thermal, chemical, and mechanical conditions). Moreover, Sections 4 and 5 emphasize the state of the art of GLACEs, from pre‐GLACE formulation developments to the comprehensive discussion sustaining the thermal conversion of miniaturized 3D GLACE replicas. Yet, TPL‐fabricated GLACEs must surpass critical challenges to bridge toward the micro‐optical industry, as remarked in this work and among the literature.^[^ ^24^, ^103^, ^126^ ^]^ For examples, a present limitation encloses the availability of tailored inorganic precursors, where some metal‐organic monomers (e.g., zirconium acrylate) are being discontinued from vendors or insufficient surface‐protected NP's prospects are found, limiting the exploration of novel GLACE candidates with higher chemical complexity^[^ ^133^ ^]^ or optical functionality relevancy. From this perspective, an effort should be made to design new synthetic molecules for GLACE formulations. In addition, parallel obstacles include achieving geometry‐shrinking fidelity to guarantee accurate manufacturing reproducibility. The  expansion of our understanding of GLACE's thermodynamic equilibrium during thermal conversion is an asset. The latter should encompass the understanding of crystal growth formation kinetics. New knowledge can be generated by collectively gathering such understanding and further being applied to tune defect characteristics (sections 6 and 7) in GLACEs to maximize optical performance.

The possibility of multi‐material printing of GLACEs is facilitated (section 7) by integrating fluidic handling systems with TPL. However, material‐based considerations like inorganic loading of the photoresin and precursor compatibility with TPL should be explored to the fullest extent possible to enable the production of mechanical and chemical‐resistant optical‐grade GLACE components. Whether using single or multiple printing steps, it is essential to use advanced characterization methods. These methods will enable industry and researchers to thoroughly understand GLACE's general characteristics or, if necessary, its more fundamental properties. This includes integrating advanced methods to shed light on the dynamic properties of GLACE microarchitecture formation upon annealing at micrometer and nanometer scales. While the techniques outlined in section 8 are substantial, other methods might also be crucial depending on the scientific question and the end application of the optical components. By leveraging these sophisticated tools, we can pave the way for significant glass and ceramic engineering advancements, ensuring that GLACEs meet their full potential in optical functionality, like micro‐optical components (section 9) and integrated optical devices (section 10).

## Conflict of Interest

The authors declare no conflict of interest.

## Author Contributions

J.A.‐D. and C.R.‐A. contributed equally to this work. J.A.‐D., C.R.‐A., and D.J. performed writing – original draft. A.S.‐A. performed writing – original draft and review & editing. All authors contributed to the review & editing of the final draft.
